# Operational epidemiology of the early phase of the 2026 Bundibugyo virus disease outbreak in the Democratic Republic of the Congo and Uganda

**DOI:** 10.1371/journal.pgph.0006680

**Published:** 2026-08-27

**Authors:** Tambe Elvis Akem, Eta Calvin Oben

**Affiliations:** 1 Independent Consultant, Yaoundé, Cameroon; 2 Independent Consultant, Barcelona, Spain; Washington State University, UNITED STATES OF AMERICA

## Abstract

The 2026 Bundibugyo virus disease outbreak in the Democratic Republic of the Congo (DRC) and Uganda occurred in a fragile cross-border setting marked by delayed recognition, diagnostic uncertainty, insecurity, population mobility, community mistrust, and healthcare-worker exposure. We conducted a retrospective operational epidemiology study using official situation reports, public health declarations, ministry statements, WHO and Africa CDC updates, cross-border communiqués, and selected partner statements. This analysis reflects publicly available data from the early outbreak period, with the primary DRC data lock on 27 May 2026 and Uganda and regional triangulation through 29 May 2026. We reconstructed the early timeline, described epidemiological and geographic progression, quantified response indicators, and applied a transparent health-zone prioritization framework. The earliest documented symptom onset was 24 April 2026. The Institut National de Recherche Biomédicale (INRB) detected non-Zaire ebolavirus in eight of 13 samples on 14 May, giving a 20-day symptom-onset-to-first-laboratory-detection interval; species-level Bundibugyo virus confirmation and official declaration followed on 15 May. By the DRC data lock on 27 May, 125 confirmed cases and 17 confirmed deaths had been reported, giving a confirmed case fatality ratio of 13.6%. DRC also reported 906 suspected cases and 223 suspected deaths, giving a suspected fatality proportion of 24.6%. Confirmed cases expanded from three to 13 health zones across three provinces within 12 days. Ituri province accounted for 110 confirmed cases (88.0%); Bunia, Rwampara, and Mongbwalu health zones together contributed 90 cases (72.0%). Uganda reported nine confirmed cases and one death by 29 May. Operational pressure included 2,635 listed contacts in DRC, 436 contacts under follow-up in Uganda, and 126 of 774 collected samples (16.3%) pending testing in DRC. Healthcare workers represented 16 of 125 DRC confirmed cases (12.8%) and at least three of nine Uganda cases (33.3%). Applying the health-zone prioritization framework, Rwampara, Mongbwalu, and Bunia were classified as higher priority health zones. Excluding confirmed burden did not change any health-zone tier; data classification confidence was high for 10 zones and moderate for three. Within this early operational snapshot, outbreak visibility reflected both transmission and operational factors, particularly detection delay, laboratory backlog, cross-border movement, healthcare-linked exposure, and community trust or security incidents. The framework provides a transparent basis for rapid decision support during an outbreak’s early phase by directing surveillance, laboratory, case-management, infection prevention and control, contact-tracing, and community-engagement resources toward health zones where multiple operational risks converge.

## Introduction

Bundibugyo virus disease (BVD) is a form of Ebola disease caused by Bundibugyo virus (BDBV), a distinct ebolavirus species first characterized after a haemorrhagic fever outbreak in western Uganda in 2007 [1,2].

The 2026 outbreak in the Democratic Republic of the Congo (DRC) and Uganda represents a major public health challenge because it involves Bundibugyo ebolavirus, a non-Zaire ebolavirus species for which no licensed vaccine or specific therapeutic is currently available [1,3,4]. Operationally, the response has unfolded in a fragile cross-border setting marked by insecurity, displacement, mining-related mobility, health-system strain, and population movement linking eastern DRC with Uganda and South Sudan [5–7]. The DRC Ministry of Health officially declared the country’s 17th Ebola disease outbreak on 15 May 2026 after laboratory confirmation of BVD in affected health zones in Ituri Province [8].

The event rapidly reached international concern. On 17 May 2026, the World Health Organization (WHO) Director-General determined that the outbreak constituted a Public Health Emergency of International Concern (PHEIC) under the International Health Regulations (IHR) [9]. The first meeting of the IHR Emergency Committee was convened on 19 May, and temporary recommendations were issued on 22 May to address surveillance, laboratory capacity, cross-border coordination, infection prevention and control (IPC), safe and dignified burials, and risk communication [10]. WHO updates published on 21 and 29 May described the evolution of the outbreak in DRC and Uganda, including imported and healthcare-linked cases in Uganda [4,11].

The outbreak was not first visible through laboratory confirmation. Earlier public reports and preliminary investigations documented unusual deaths, severe febrile illness, healthcare-worker deaths, and clustered illness in Mongbwalu and Rwampara health zones before confirmation of BVD [12,13]. WHO reported that the first currently known suspected case was a healthcare worker (HCW) with symptom onset on 24 April 2026, while initial testing and confirmation occurred later in May [3,9]. Africa CDC called for urgent regional coordination on 15 May 2026 because of the risk of spread from Ituri Province [14]. On 18 May 2026, Africa CDC declared the outbreak a Public Health Emergency of Continental Security (PHECS) [5]. Uganda confirmed imported cases from DRC and activated surveillance, screening, isolation, IPC, and risk communication measures [4,11,15].

Publicly released national, regional, and international outbreak updates provided a timely opportunity to examine how early signals, diagnostic confirmation, geographic spread, health-worker exposure, and response constraints shaped the first phase of the outbreak [4,12,13,16,17].

This outbreak occurred in a context where technical response capacity was shaped by social and security realities, including displacement, mining-related mobility, funeral practices, linguistic diversity, insecurity, and mistrust toward response actors [6]. These factors influenced local engagement and the practical implementation of surveillance, IPC, risk communication, and safe care.

Independent early modelling by Imperial College London suggested that the true scale of the outbreak may have been substantially larger than confirmed counts during the early phase, with approximately 400–800 cases potentially having occurred in DRC by 17 May 2026 and values above 1,000 not excluded under uncertainty [7]. The present study complements the modelling by focusing on operational epidemiology: what public data reveal about detection delay, laboratory and surveillance pressure, contact tracing, IPC vulnerability, cross-border risk, and decision priorities. In the early phase of high-consequence outbreaks in fragile settings, what becomes visible through public data, including confirmed case counts, case fatality ratios, and growth trajectories, is a joint product of true transmission dynamics and operational factors such as detection capacity, laboratory throughput, reporting completeness, and access. This study treats confirmed epidemiological indicators not as direct measures of transmission but as imperfect observational proxies shaped by the operational system that generates them; this framing motivates the parallel analysis of detection gaps, the distinction between confirmed affected and operationally monitored health zones, and a health-zone operational prioritization framework alongside the epidemiological description that follows. The aim of this study was to reconstruct the early trajectory and operational response bottlenecks of the 2026 BVD outbreak in DRC and Uganda using publicly available data, and to generate a concise decision-support framework for outbreak control prioritization.

## Materials and methods

### Ethics statement

This study used publicly available, aggregated outbreak data and official public documents. No individual-level identifiable data were accessed or analysed. The study did not involve direct interaction with patients, contacts, HCWs, responders, or community members, and informed consent was therefore not applicable. Ethical approval was not sought because the analysis was based exclusively on public, aggregated, non-identifiable data.

### Study design

We conducted a retrospective public-data operational epidemiology study using mixed quantitative and structured documentary analysis. The quantitative component used aggregated health-zone, provincial, country-level, and regional indicators extracted from official public documents. The structured document review component coded response bottlenecks across outbreak response pillars, including coordination, surveillance, laboratory, contact tracing, case management, IPC and water, sanitation, and hygiene (WASH), safe and dignified burials, risk communication and community engagement, logistics, security, mobility, cross-border coordination, health-worker risk, and data quality.

### Study setting and period

The primary setting was the DRC, with emphasis on affected health zones in Ituri, North Kivu, and South Kivu provinces. Uganda was included for confirmed imported cases, healthcare-linked transmission, contact management, and cross-border preparedness. South Sudan was considered only as a preparedness and cross-border risk context because no official case had been documented in South Sudan by the analysis lock, 27 May 2026. The primary analysis period was 24 April 2026 to the analysis-lock date, 27 May 2026. The start date reflects the earliest documented symptom-onset information in public outbreak materials. The principal DRC data lock used official DRC data reported through 27 May 2026 [16]. Uganda and regional values were triangulated with WHO updates and Uganda Ministry of Health (MOH) statements through 29 May 2026 [4]. These data locks were retained to preserve the study’s focus on the early operational phase and were not updated to reflect the subsequent evolution of the outbreak.

### Data sources

Data were extracted from public, aggregated, non-identifiable sources. Primary DRC data were obtained from situation reports issued by the Centre des Opérations d’Urgence de Santé Publique de la République démocratique du Congo (COUSP-RDC), within the Institut National de Santé Publique (INSP) [16]. Uganda data came from Uganda MOH public statements and WHO Disease Outbreak News [4,15]. Regional triangulation and governance milestones were extracted from WHO updates, Africa CDC statements, and cross-border coordination documents [4,11,14]. Selected official partner statements were used only when they documented operational incidents relevant to outbreak control, such as treatment-site security events [17]. Unverified social media posts, individual patient identifiers, unsupported media claims, and non-public operational datasets were excluded. Where social media was mentioned in official reports as part of the outbreak signal, it was treated only as a documented route of alert generation, not as an independent data source.

### Source hierarchy and reconciliation

When public sources reported different figures for the same reporting date, DRC COUSP/INSP situation reports were prioritized for DRC data [16]. Uganda MOH statements and WHO Disease Outbreak News were prioritized for Uganda data [4,15], depending on the variable and date. WHO and Africa CDC updates were used for regional triangulation, governance milestones, and interpretation when national values were unavailable. Official partner statements were used for operational incidents not captured in national or WHO reports.

If a later official situation report revised an earlier cumulative figure, the later official value was treated as the corrected value, while the earlier value remained documented in the internal discrepancy log. Values reported as “not determined,” “not reported,” or “not available,” as well as values classified as not assessable (NA) during analysis, were treated as missing and were not assigned a value of zero. A value of zero was used only when the source explicitly reported zero or clearly documented the absence of the relevant signal.

### Unit of analysis

Data were analysed by health zone and reporting date. When daily health-zone data were unavailable, values from each available situation report were used as the corresponding observation for that reporting period. Where health-zone information was unavailable, data were coded at province, country, or regional level. The document review unit was the source document and the response-pillar segment within each document.

### Two-denominator framework

The analysis used two denominators. Confirmed affected health zones were defined as zones with at least one laboratory-confirmed case in official situation reports and were used for confirmed burden, confirmed case fatality ratio (CFR), and geographic spread analyses. Operationally monitored zones were defined as zones with documented epidemiological or response activity, including suspected cases, deaths, contacts, alerts, IPC activities, or field follow-up, and were used for operational indicators. By the 27 May data lock, 13 DRC health zones across Ituri, North Kivu, and South Kivu had confirmed cases. Zones with suspected cases, contacts, or operational follow-up but no confirmed case, including Bambu, Karisimbi, and Kyondo, were retained as operationally monitored but were not classified as confirmed affected zones.

### Handling of unassigned confirmed cases

Country totals included all laboratory-confirmed cases regardless of geographic assignability. In the DRC data reported through 27 May 2026, six confirmed cases were recorded as “Echantillons sans fiche” (samples without forms). These cases were included in the DRC national confirmed total but excluded from health-zone-level burden analysis because they could not be attributed to a specific health zone. They were classified as unassigned in the analysis workbook.

### Variables and indicators

Core epidemiological variables included suspected cases, confirmed cases, deaths among suspected or confirmed cases, recovered cases, affected health zones, affected provinces, HCW infections or deaths, imported cases, and medically evacuated cases. Probable cases were not analysed as a core category because no consistent numerical probable-case classification was reported. Core response variables included alerts, laboratory testing, isolation, contacts, treatment or isolation capacity, IPC and WASH gaps, security incidents, misinformation, community resistance, safe and dignified burials, and cross-border response actions.

Derived indicators included confirmed CFR, suspected case fatality proportion, alert investigation rate, sampling proportion, isolation coverage, laboratory positivity, laboratory backlog, contacts per confirmed case, contact follow-up rate where available, and newly affected confirmed health zones per reporting period. Indicators were calculated only when numerators and denominators were sufficiently clear.

### Detection gap analysis

The primary outcome was the operational detection gap, defined as the elapsed time between successive milestones in outbreak recognition and response. Separate intervals were calculated between earliest documented symptom onset, first community signal, official notification, laboratory confirmation, official declaration, Uganda confirmation, WHO PHEIC determination, Africa CDC PHECS declaration, and cross-border coordination milestones. Date differences were calculated using calendar-day intervals.

### Epidemiological trajectory and geographic spread

The analysis described confirmed cases, confirmed deaths, suspected cases, suspected deaths, contacts, and affected health zones across reporting periods. DRC cumulative trajectories were based on COUSP/INSP situation reports. Uganda progression was summarized separately using Uganda MOH and WHO Disease Outbreak News data. Geographic analysis ranked confirmed affected health zones at the DRC data lock and reported operationally monitored but non-confirmed zones separately.

### Laboratory, contact tracing, and IPC indicators

Laboratory analysis summarized samples collected, samples analysed, positive results, positivity, and backlog where available. Contact tracing analysis summarized listed contacts, contacts per confirmed case, and country-specific contact-tracing progression. IPC analysis separately examined strict HCW confirmed cases and broader healthcare-linked or occupational exposure. Uganda HCW cases were analysed alongside DRC HCW cases to assess cross-border IPC vulnerability, while avoiding pooled rates unless clearly labelled as descriptive minimums.

### Health-zone operational prioritization framework

A pragmatic health-zone operational prioritization (HZ-OP) framework for confirmed affected health zones was used to classify zones into lower, moderate, or higher priority for response escalation. The framework comprised two conceptually distinct groups of indicators. Epidemiological-operational domains captured documented disease burden and outcome pressure as observed through the surveillance and response system: confirmed burden, suspected mortality burden, and case fatality proportion based on suspected or confirmed cases. Purely operational domains captured response workload, implementation gaps, and potential amplification conditions: contact network size, contact coverage gap, IPC or HCW risk, security or community-trust risk, and mobility or cross-border relevance.

Each domain was scored from 0 to 2, giving a minimum total score of 0 and a maximum total score of 16. Zones were classified as lower priority (0–4), moderate priority (5–8), or higher priority (9–16). The full scoring rubric is provided in S1 Table. Confirmed burden was retained despite reflecting both underlying transmission and the operational system through which infections are detected, tested, and reported, because it also represents documented caseload and immediate resource demand for isolation, treatment, laboratory support, and contact investigation. It was not interpreted as an unbiased measure of underlying transmission intensity. The case fatality proportion domain was designed as a phase-adaptive measure using two independently assessed pathways. The suspected-fatality pathway was calculated as suspected deaths divided by suspected cases where both values were available; explicitly reported zero suspected cases and zero suspected deaths were treated as an assessable absence of a suspected-fatality signal without calculating a proportion. The confirmed-CFR pathway was calculated as confirmed deaths divided by confirmed cases and was assessable only when zone-level confirmed deaths were available and at least five confirmed cases were reported, reducing instability from small denominators. Where both pathways were assessable, the higher domain score was retained. During the early phase of an outbreak, suspected cases and deaths may be more consistently available at health-zone level because testing capacity, laboratory throughput, and outcome classification remain incomplete; as testing and confirmed-outcome reporting mature, the confirmed-CFR pathway becomes increasingly applicable. Numerical thresholds for the domains applied in the primary analysis were empirically calibrated to the observed health-zone data distribution at the analysis lock, with rounded cut-points selected to distinguish limited, intermediate, and upper-range documented signals while remaining operationally interpretable. The confirmed-CFR pathway was retained as a phase-adaptive alternative using pre-specified thresholds (20% and 40%, requiring at least five confirmed cases); these thresholds were not empirically derived from the present health-zone distribution and require reassessment when replicated in a later outbreak phase or setting with zone-level confirmed-death reporting. Equal weighting was used across all eight domains to maintain transparency and reproducibility and because no validated evidence was available to support differential weights for this outbreak; equal weighting should not be interpreted as evidence that all domains have equivalent causal or predictive importance.

A data classification confidence category was assigned to each health-zone score according to the completeness of publicly available evidence across the eight domains. Confidence was classified as high when all eight domains were assessable, moderate when five to seven domains were assessable, and low when fewer than five domains were assessable. A domain was considered assessable when available sources provided sufficient information to assign a score of 0, 1, or 2; explicitly reported zero values and clearly documented absence of a qualifying signal were treated as available information, whereas missing, unreported, not determined, insufficiently disaggregated, or otherwise non-assessable information was treated as unavailable and did not contribute a score. Data classification confidence reflects the completeness of the public evidence supporting a classification and should not be interpreted as statistical uncertainty, predictive validity, or confirmation of the accuracy of the underlying data. The framework was not designed or presented as a validated prediction model, but as a transparent rapid decision-support tool for summarizing multiple public outbreak signals at a defined data lock. It was intended to identify where operational escalation may be required, not to estimate underlying transmission risk, forecast future cases, or replace field investigation.

All scores represent public information available at the 27 May 2026 DRC data lock and should not be interpreted as current classifications beyond that date.

### Growth analysis

Confirmed case growth was described visually and narratively. A log-linear estimate of cumulative confirmed cases over time was calculated only as an exploratory descriptive analysis. Formal Poisson or negative binomial regression was not used because the confirmed case time series was short and strongly affected by laboratory throughput and testing decisions. Confirmed case deceleration was not interpreted as transmission control unless supported by independent operational evidence.

### Sensitivity analyses

Sensitivity analyses included restriction to confirmed cases only, alternative definitions of the detection interval, exclusion of unassigned confirmed cases from health-zone rankings, assessment of the discrepant suspected-death totals reported for 23 May 2026, and reclassification of health zones after removing the confirmed-burden domain from the operational prioritization framework.

For the alternative detection-interval analysis, first laboratory detection on 14 May 2026 was retained as the fixed endpoint. The starting point was varied between earliest documented symptom onset on 24 April, first community signal on 5 May, and official alert notification on 8 May, producing three interval estimates to the same detection date.

For the confirmed-burden-excluded analysis, the 13 confirmed affected health zones were rescored using the remaining seven domains. The original absolute priority cut-points were retained without recalibration: 0–4 for lower priority, 5–8 for moderate priority, and 9 or more for higher priority. This provided a conservative assessment of whether the confirmed-burden domain was necessary for any health zone to cross a priority threshold.

### Reporting guideline and software

The study was reported in accordance with STROBE guidance [18]; the completed checklist is provided in the Supporting Information as S1 Checklist. Data cleaning, validation, analysis, and visualization were performed using Python.

## Results

### Early detection and governance timeline

The earliest documented symptom onset among confirmed cases was 24 April 2026 in a HCW in Rwampara health zone, Ituri Province. A community alert via social media, noting unusual deaths of unknown cause in Mongbwalu health zone, was documented on 5 May 2026. An official alert notification was made at provincial level on 8 May 2026. Field investigations on 12–13 May documented severe febrile illness, deaths, and health-worker involvement before Ebola-specific laboratory confirmation was available [12,13]. Initial sample testing was negative for Ebola Zaire, dengue, rotavirus, cholera, malaria, Yersinia pestis, mpox, and COVID-19 [13,16].

On 14 May 2026, DRC INRB in Kinshasa analysed 13 blood samples from Rwampara Health Zone; eight tested positive for a non-Zaire ebolavirus. On 15 May, BVD was confirmed, with sequencing identifying Bundibugyo virus, and the DRC Ministry of Health officially declared the country’s 17th Ebola outbreak the same day [3,8].

**Fig 1:**
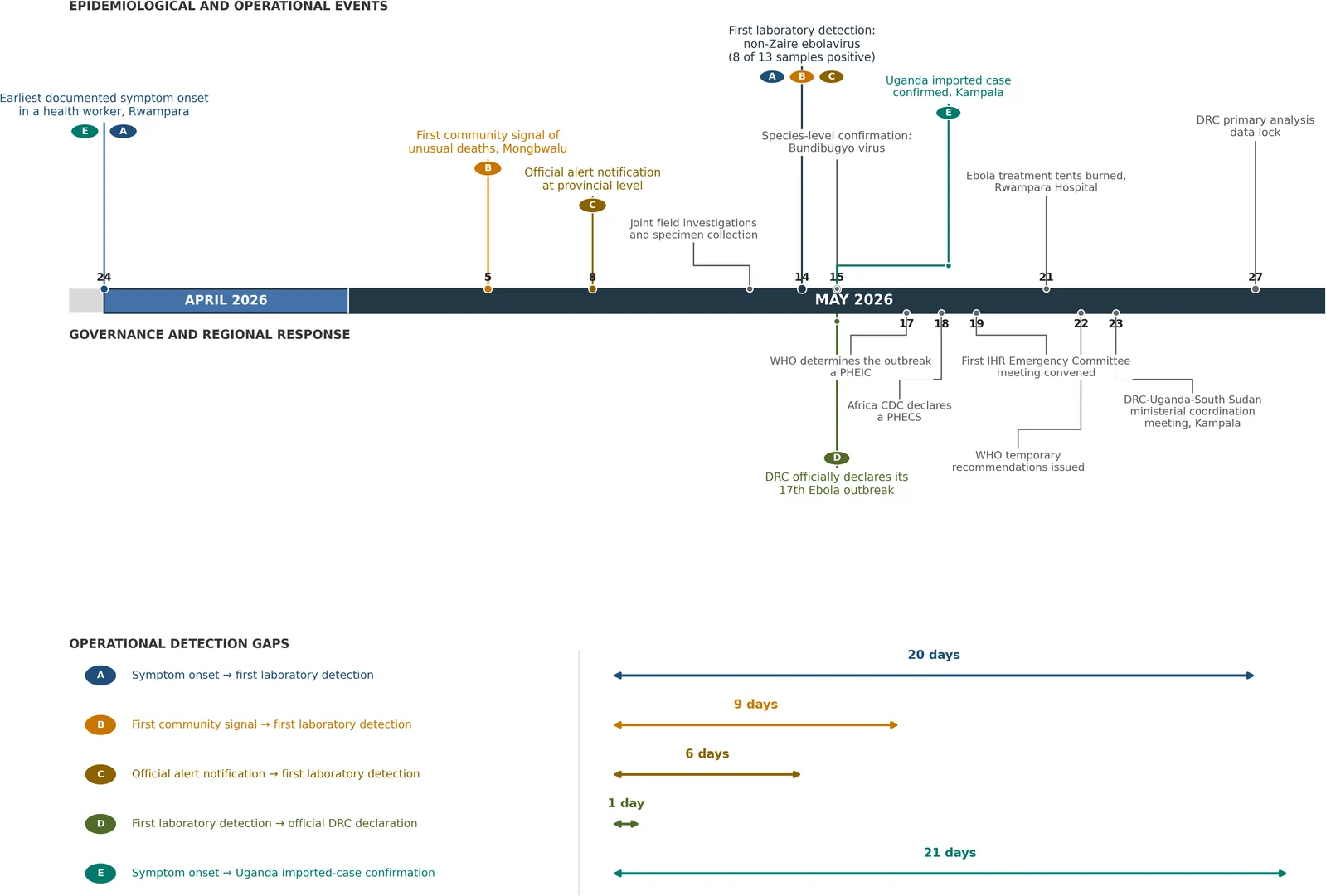
Early detection, cross-border, and governance timeline for the 2026 Bundibugyo virus disease outbreak in DRC and Uganda. The upper timeline presents selected epidemiological, operational, and governance milestones from the earliest documented symptom onset on 24 April 2026 to the DRC primary analysis data lock on 27 May 2026. First laboratory detection of a non-Zaire ebolavirus occurred on 14 May, followed by species-level confirmation of Bundibugyo virus, confirmation of the imported Uganda case, and official DRC declaration on 15 May. The lower panel shows five operational detection gaps in elapsed calendar days: Gap A, symptom onset to first laboratory detection; Gap B, first community signal to first laboratory detection; Gap C, official alert notification to first laboratory detection; Gap D, first laboratory detection to official DRC declaration; and Gap E, symptom onset to Uganda imported-case confirmation. Gap arrows are proportional to elapsed duration and are presented schematically.

Five detection-gap intervals were calculated. The earliest documented symptom onset was 24 April 2026, and first laboratory detection occurred on 14 May, giving a 20-day symptom-onset-to-first-laboratory-detection interval (Gap A, Fig 1). The interval from the first community signal on 5 May to first laboratory detection was 9 days (Gap B), while the interval from official alert notification on 8 May to first laboratory detection was 6 days (Gap C). Following first laboratory detection, official DRC declaration occurred within 1 day (Gap D). Uganda confirmed an imported case on 15 May 2026, the same day as the DRC declaration, through retrospective testing of a stored sample from a Congolese man who had sought care in Kampala on 11 May and died on 14 May. The interval from earliest documented symptom onset in DRC (24 April) to Uganda confirmation (15 May) was 21 days (Gap E).

In terms of governance escalation, the WHO Director-General determined the outbreak to be a PHEIC under the IHR on 17 May 2026, two days after the DRC declaration [9]. Africa CDC declared a PHECS on 18 May 2026 [5]. WHO convened the first meeting of the IHR Emergency Committee on 19 May, and temporary recommendations were issued on 22 May [10]. A high-level cross-border coordination meeting involving DRC, Uganda, and South Sudan was held in Kampala on 23 May 2026 [14]. The complete timeline from early signal to data lock is shown in Fig 1.

### Epidemiological trajectory and geographic spread

#### DRC confirmed and suspected case progression.

**Fig 2:**
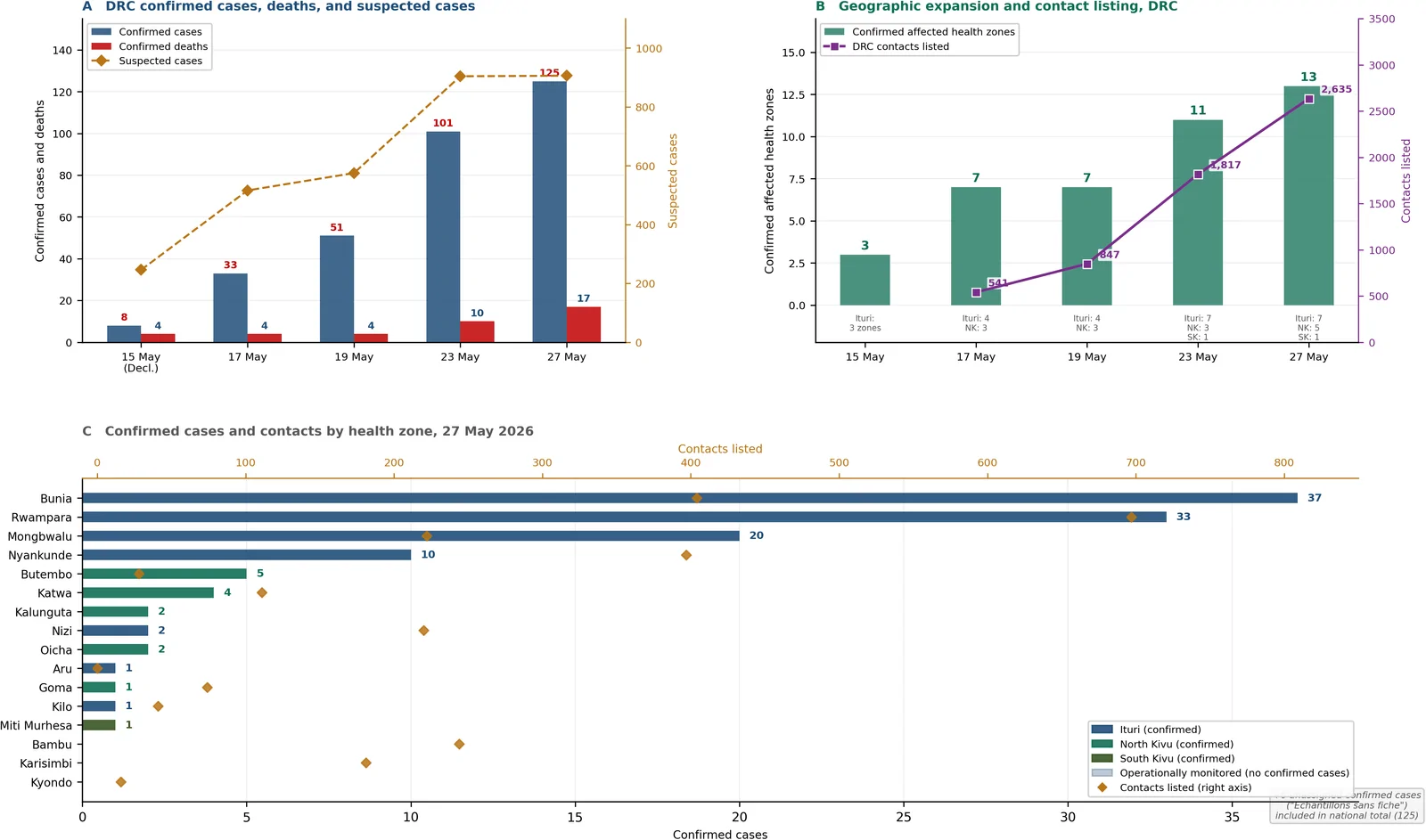
Outbreak progression and geographic spread of the 2026 Bundibugyo virus disease outbreak in DRC. Panel A: DRC cumulative confirmed cases, confirmed deaths, and suspected cases at five reporting timepoints. Panel B: confirmed affected DRC health zones and contacts listed over time. Panel C: confirmed cases and contacts by health zone using DRC data reported through 27 May 2026. Faded bars represent operationally monitored zones with no confirmed case. Six unassigned confirmed samples are included in the national DRC total of 125 but are not shown as a health-zone bar. No contact marker is shown for health zones where the contact count was missing or not determined.

At official DRC declaration on 15 May 2026, eight laboratory-confirmed cases and four confirmed deaths had been reported, alongside 246 suspected cases and 80 suspected deaths [3,8]. By 27 May, confirmed cases had increased to 125 and confirmed deaths to 17, while suspected cases and suspected deaths had reached 906 and 223, respectively (Fig 2A). Contacts listed in DRC increased from 541 on 17 May to 2,635 on 27 May, a 4.9-fold increase over 10 days (Fig 2B). The confirmed CFR was 50.0% at declaration (4/8), declined to 7.8% on 19 May as testing expanded, and reached 13.6% (17/125) by 27 May. This trajectory should be interpreted cautiously because confirmed cases and deaths were influenced by testing throughput, case ascertainment, and backlog. The suspected fatality proportion was 24.6% (223/906) on 27 May, but this should not be interpreted as a disease-specific population CFR because suspected cases include non-confirmed illnesses.

An exploratory log-linear description of five cumulative confirmed case data points estimated a daily growth rate of 22.8% and a doubling time of 3.4 days. This estimate is descriptive only, given the short 12-day window, limited number of data points, and dependence of confirmed counts on testing and reporting processes. The slower increase between 23 and 27 May may reflect laboratory backlog or reporting constraints and should not be interpreted as evidence of reduced transmission without independent operational evidence.

#### Geographic expansion.

At declaration, three health zones in Ituri Province had confirmed cases: Mongbwalu, Rwampara, and Bunia. By 17 May, expansion to North Kivu had already occurred, with confirmed cases in Goma, Katwa, and Butembo, yielding seven confirmed affected zones across two provinces. The number of confirmed affected zones reached 11 by 23 May and 13 by 27 May, spanning Ituri (seven zones), North Kivu (five zones), and South Kivu (one zone). Three additional zones, Bambu in Ituri and Karisimbi and Kyondo in North Kivu, were operationally monitored because contacts or suspected activity were documented, but they had no confirmed cases by 27 May.

Of the 125 confirmed cases reported in DRC by 27 May, Ituri Province accounted for 110 (88.0%). Among health zones, Bunia had the highest number of recorded confirmed cases (37; 29.6% of the DRC total), followed by Rwampara (33; 26.4%), Mongbwalu (20; 16.0%), and Nyankunde (10; 8.0%). Six confirmed cases were recorded as samples without attribution forms; these were included in the national DRC total but excluded from zone-level analyses. North Kivu accounted for 14 confirmed cases, and South Kivu accounted for one confirmed case. Suspected case burden was most concentrated in Mongbwalu (339 suspected cases), which also had the highest suspected death count (88). The distribution of confirmed cases and listed contacts by health zone is shown in Fig 2C.

#### Uganda case progression and additional international case.

Uganda confirmed one imported case on 15 May 2026. Two Uganda confirmed cases, including one death, were reported as of 20 May, and 127 contacts were under follow-up as of 18 May. By 23 May, five cases had been confirmed. Two additional Ugandan HCWs at a private Kampala facility were confirmed on 25 May, and by 29 May, nine confirmed cases and one confirmed death had been reported, with 436 contacts under follow-up [4,15]. Of the nine Uganda confirmed cases, eight were in Kampala and one in Wakiso. The combined DRC-Uganda regional total was 134 confirmed cases and 18 confirmed deaths. A separately reported medical evacuation of a United States physician, exposed while providing patient care in DRC and transferred to a high-level isolation unit in Berlin, Germany, for treatment, was documented as an additional international case and was excluded from the 134 DRC-Uganda regional total [4].

### Operational response indicators

#### Surveillance and alert investigation.

Alert investigation rates remained broadly stable across the analysis period. On 18 May, 54 of 64 alerts reported in Ituri Province that day (84.4%) were investigated, and 53 of the 64 alerts (82.8%) were validated [16]. By 27 May, 98 of 118 alerts (83.1%) had been investigated, of which 51 (52.0%) were validated. The lower validation proportion may reflect the geographic expansion of surveillance, with a greater number of investigated alerts ultimately classified as inconsistent with Ebola. Of the 51 validated alerts, 35 (68.6%) had been sampled, leaving 16 (31.4%) unsampled and indicating a potential bottleneck in the pathway from alert validation to laboratory confirmation.

#### Laboratory performance and testing backlog.

By 27 May, 774 samples had been collected cumulatively, of which 648 (83.7%) had been analysed and 125 (19.3% of analysed samples) were positive. A cumulative backlog of 126 samples remained pending testing. Same-day laboratory processing was constrained at the Ituri laboratory: on 27 May, only 3 of 73 samples received (4.1%) were analysed on the reporting day. Qualitative findings also documented difficulty transporting samples to the Goma and Kinshasa laboratories. Earlier in the outbreak, all 27 samples received on the reporting day had been analysed, with 18 positive results. This comparison suggests that laboratory processing capacity did not keep pace with increasing sample volume as the outbreak expanded geographically. The backlog limits the interpretability of confirmed case trends; the slower increase in confirmed cases between 23 and 27 May should therefore be interpreted in this context, rather than as evidence of transmission reduction.

#### Contact tracing and contact burden.

DRC contacts listed grew from 541 (17 May) to 1,817 (23 May) and 2,635 (27 May). The contact-to-confirmed-case ratio increased from 16.4 (17 May) to 21.1 (27 May), consistent with active exposure listing and broad contact networks associated with health-facility and community transmission. Zone-level contact data indicated potential gaps: Aru health zone, with one confirmed case and one suspected death, recorded zero listed contacts by 27 May, and three confirmed zones (Kalunguta, Oicha, and Miti-Murhesa) had contact counts reported as missing or not determined in the extracted data. These gaps should be interpreted as potential reporting or listing limitations rather than absence of contacts. Uganda had 127 contacts under follow-up by 18 May and 436 by 29 May, a contact-to-case ratio of 48.4.

#### Case management and isolation.

By 27 May, 103 patients were reported hospitalized, of whom 92 (89.3%) were suspected and 11 (10.7%) confirmed. Bed occupancy among suspected patients was 70.1%, and bed occupancy among confirmed patients was 8.7%. Reports documented absence of standardized transit or Ebola treatment centres (ETCs) in Ituri Province and limitations in essential medicines in improvised isolation structures at the time of early field assessment. These constraints were operationally significant given that the outbreak originated in and remained concentrated in Ituri, and that emergency treatment infrastructure had not yet been fully established at the time of declaration.

#### Health-worker and healthcare-linked IPC risk.

HCW exposure was documented in both countries. Among the 125 confirmed cases reported through 27 May, 16 (12.8%) were HCWs, as reported in WHO’s 29 May updates [4]. The preliminary Rwampara investigation documented eight suspected cases among frontline health personnel within a broader line list of 27 suspected cases. IPC compliance scores of 7% at Abelkozo health centre and 34% at Mongbwalu General Referral Hospital were documented in early field assessments [12,13]. Four HCW deaths within four days before laboratory confirmation were documented in the early Mongbwalu report [13].

In Uganda, at least three of nine confirmed cases (33.3% minimum) were HCWs, comprising one Congolese HCW linked to the index case and two Ugandan HCWs at a private Kampala facility linked to an earlier confirmed case [4]. At least four of nine cases (44.4% minimum) had healthcare-linked or occupational exposure when the Ugandan driver who transported the index case is included; this individual was classified as occupationally exposed, not as a healthcare worker.

#### Security, community trust, and point-of-entry screening.

On 21 May 2026, the Alliance for International Medical Action (ALIMA) reported that two Ebola treatment tents at Rwampara Hospital were set on fire while six patients were receiving care [17]. The incident was associated with misinformation, fear, and mistrust. The early Mongbwalu report had previously documented generalized fear and rumours of mystical causation before laboratory confirmation [13]. The Ituri contextual note highlighted language diversity, displacement, mining-related mobility, funeral practices involving body handling, and a narrative in which some community members perceived financial benefit to response actors from the Ebola response [6]. These dynamics were documented as operational risk signals.

At DRC points of entry and points of control, 14,530 of 14,662 travelers were screened (99.1%) and 14,314 received handwashing facilitation (97.6%) by 27 May. Qualitative sources consistently noted that formal point-of-entry data do not capture informal transit routes, including mining transport corridors and non-standard travel routes used by some Uganda-confirmed cases [4,6,14].

### Health-zone operational prioritization

**Fig 3:**
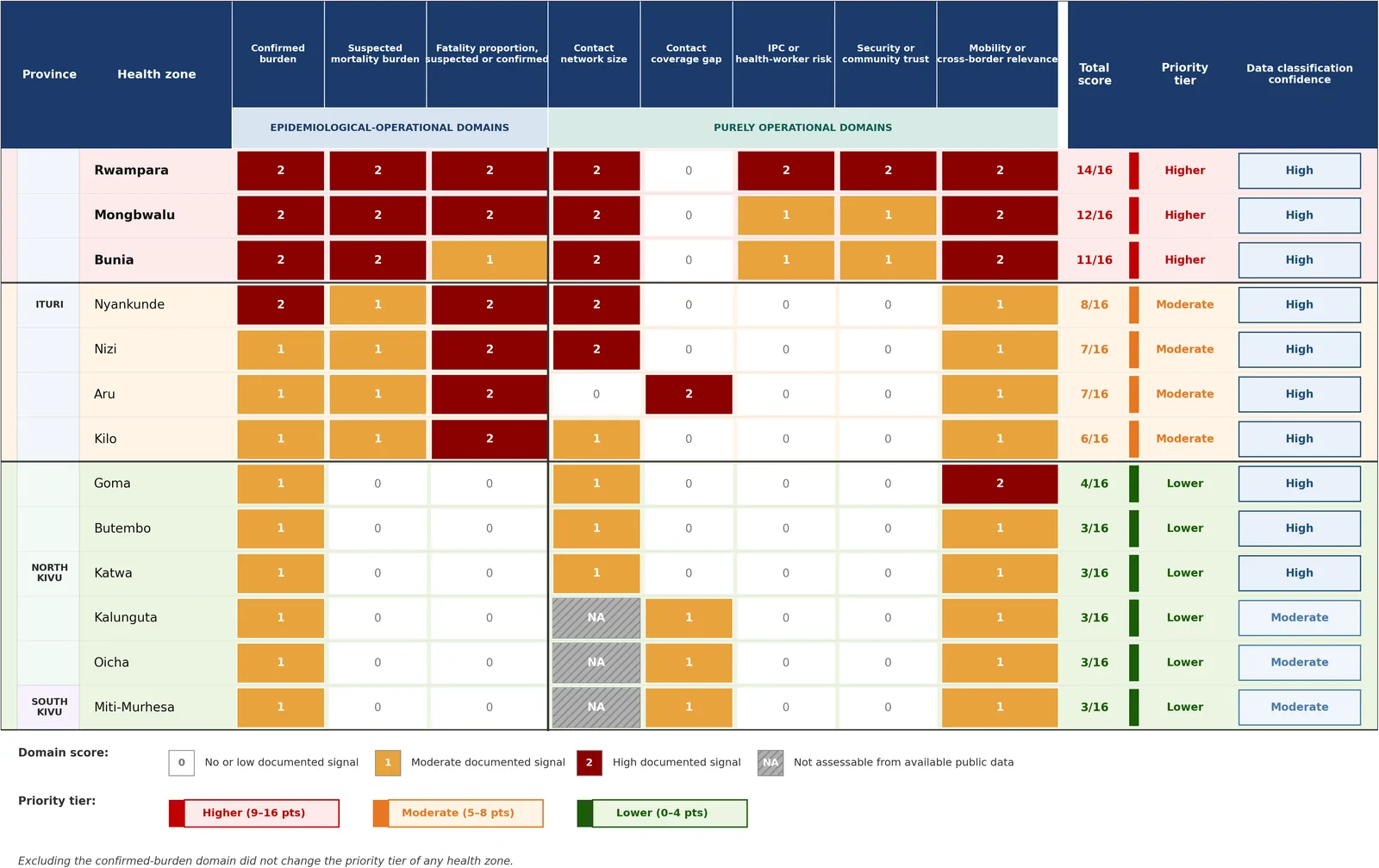
Operational prioritization of confirmed affected health zones in DRC as of 27 May 2026.

Applying the health-zone operational prioritization (HZ-OP) framework to public information available at the 27 May 2026 data lock (Fig 3), three health zones were classified as higher priority: Rwampara (14/16), Mongbwalu (12/16), and Bunia (11/16). Rwampara combined high confirmed and suspected burden, the largest listed contact network, documented health-worker and IPC risk, the treatment-site security incident, and cross-border relevance. Mongbwalu combined high suspected burden, high suspected mortality, early community mortality signals, IPC concerns, health-worker deaths, mining-related mobility, and a large contact network. Bunia had the highest confirmed case count, a large contact network, and its role as the Ituri provincial capital and transport hub.

Four Ituri health zones were classified as moderate priority: Nyankunde (8/16), Nizi (7/16), Aru (7/16), and Kilo (6/16). Aru required particular attention because it had confirmed activity and suspected mortality but no listed contacts by the data lock, suggesting a possible contact-listing or reporting gap. Six health zones were classified as lower priority: Butembo, Katwa, Kalunguta, Oicha, Goma, and Miti-Murhesa (3–4 of 16). Goma and Miti-Murhesa had confirmed cases but remained lower priority because few additional operational stress indicators were publicly documented by 27 May. However, Goma remained a strategic surveillance concern because of its population size, mobility profile, and security context.

Data classification confidence was high for 10 of 13 health zones and moderate for the remaining three (Kalunguta, Oicha, and Miti-Murhesa), reflecting incomplete public reporting of contact data in these zones; no classification had low confidence. In a sensitivity analysis excluding the confirmed-burden domain and retaining the original absolute classification thresholds, all 13 health zones retained their original priority tier (S2 Table). Rwampara, Mongbwalu, and Bunia remained higher priority under this seven-domain analysis, indicating that their classifications reflected convergence across multiple epidemiological-operational and purely operational domains rather than confirmed burden alone. Within the early-phase dataset, the observed priority tiers were unchanged after exclusion of the confirmed-burden domain.

The matrix summarizes eight domains grouped into epidemiological-operational domains (confirmed burden, suspected mortality burden, and fatality proportion based on suspected or confirmed outcomes) and purely operational domains (contact network size, contact coverage gap, IPC or health-worker risk, security or community-trust risk, and mobility or cross-border relevance). Each assessable domain was scored from 0 to 2, with higher values indicating greater documented operational pressure. NA indicates that a domain was not assessable from the available public data and is distinct from an explicitly supported score of 0. Total scores were classified as lower priority (0–4), moderate priority (5–8), or higher priority (9–16). Data classification confidence reflects the completeness of the public information supporting each classification: high when all eight domains were assessable, moderate when five to seven domains were assessable, and low when fewer than five domains were assessable. Confidence reflects data completeness and not statistical uncertainty, predictive validity, or accuracy of the underlying data. The framework is descriptive and was not designed as a validated prediction model. Excluding confirmed burden did not change the priority tier of any health zone (S2 Table).

### Sensitivity analysis

Five sensitivity checks were conducted. First, in a confirmed-only analysis, the geographic distribution and zone-level burden findings were unchanged: Ituri remained the dominant province, and Bunia, Rwampara, Mongbwalu, and Nyankunde health zones had the highest numbers of recorded confirmed cases.

Second, a discrepancy in the number of suspected deaths reported for 23 May was evaluated. The national summary on the report’s cover page listed 119 suspected deaths, whereas summing the health-zone rows in the detailed table yielded 220 suspected deaths. Using the same denominator of 904 suspected cases, these values produced suspected fatality proportions of 13.2% (119/904) and 24.3% (220/904), respectively. The health-zone table values were used for health-zone-level analyses, while the cover-page value was retained in the national time-series summary to preserve consistency with the officially reported national total. Third, alternative detection-gap definitions were assessed while holding first laboratory detection on 14 May as the fixed endpoint. Using the first community signal on 5 May gave a 9-day interval, while using official alert notification on 8 May gave a 6-day interval.

Fourth, excluding the six unassigned confirmed cases from health-zone-level analyses did not affect zone rankings, geographic distribution, or the primary findings. These cases were retained in the national and regional totals.

Fifth, excluding the confirmed-burden domain reduced health-zone scores but did not change the priority tier of any of the 13 confirmed affected health zones when the original absolute cut-points were retained (S2 Table).

## Discussion

### Principal findings

This operational epidemiology study reconstructs the early phase of the 2026 BVD outbreak using public data through 27 May 2026 for DRC and 29 May 2026 for Uganda and regional triangulation, and highlights four key findings. It represents an early snapshot, not a current or complete assessment of outbreak burden, spread, or response performance. First, laboratory confirmation occurred 20 days after the earliest documented symptom onset, whereas official declaration followed within 1 day of confirmation, indicating that the main delay occurred before laboratory confirmation. Second, the outbreak expanded rapidly from three confirmed affected health zones at declaration to 13 health zones across three DRC provinces by 27 May. Third, publicly available data documented substantial operational pressure, including laboratory backlog, expanding contact-tracing burden, HCW and healthcare-linked exposure in both DRC and Uganda, and security or trust-related incidents affecting response operations. Fourth, the health-zone operational prioritization framework identified Rwampara, Mongbwalu, and Bunia as higher-priority zones because they combined high burden, large contact networks, IPC concerns, mobility, and security or community trust risks at data lock.

### Interpreting confirmed case trends and detection delay

Confirmed case counts reflected both transmission and operational visibility, including case recognition, care-seeking, sample transport, laboratory throughput, testing capacity, and data harmonization. The apparent deceleration during the final reporting interval should therefore not be interpreted as transmission control without supporting evidence from contact follow-up, timely isolation, declining alert positivity, and reduced high-risk exposures. The 1-day interval between laboratory confirmation and official declaration indicates rapid action once confirmation was available. However, the longer interval from symptom onset and early community signals to confirmation underscores the need for earlier recognition of unusual deaths, healthcare-worker clusters, and severe febrile illness. Because early BVD symptoms are non-specific and overlap with other endemic illness [1,2], community-based and event-based surveillance are essential in remote, insecure, and hard-to-access settings to rapidly detect, verify, escalate, and link such signals to specimen collection [19].

### IPC and healthcare-worker exposure as a cross-border vulnerability

HCW exposure was not confined to the initial DRC outbreak area. In DRC, 16 confirmed cases among HCWs were reported by WHO by 29 May [4]. In Uganda, at least three of nine confirmed cases were HCWs, and at least four had healthcare-linked or occupational exposure when the driver linked to the index case is included [4,15]. This supports the interpretation that IPC vulnerability extended across the border into receiving facilities, especially where undiagnosed cases or cases with delayed diagnosis presented before BVD was recognized. This finding should not be framed as a failure of individual facilities. It reflects a predictable risk in high-consequence outbreaks: patients may present first to facilities that are not yet fully protected for the pathogen, particularly when early symptoms are non-specific and the outbreak has not yet been confirmed. A systematic review of filovirus outbreaks found that HCW infections were associated with inadequate personal protective equipment (PPE), exposure to patients with unrecognized Ebola or Marburg disease, and gaps in IPC implementation [20]. Evidence from Ebola IPC implementation research in Sierra Leone also shows that IPC effectiveness depends on training, supplies, facility-level organization, and trust between HCWs, communities, and response systems [21].

### Health-zone operational prioritization and public health decision-making

The HZ-OP framework should be interpreted as a decision-support tool, not a prediction model. Its value lies in integrating multiple public signals, including burden, mortality, contacts, IPC risk, security or trust concerns, and mobility, into an interpretable prioritization structure. Rwampara, Mongbwalu, and Bunia emerged as higher-priority zones, but for different operational reasons. This distinction matters because response escalation should not be uniform across zones: Rwampara requires strong IPC, HCW protection, and trust restoration; Mongbwalu requires intensified community-based surveillance, mortality investigation, and engagement with mining-linked mobile populations; and Bunia requires strengthened urban surveillance, referral coordination, laboratory linkage, and contact-tracing capacity. The inclusion of confirmed burden as a domain requires careful interpretation because reported case counts reflect both underlying transmission and the operational system through which infections are detected, tested, and reported. Its inclusion was therefore not intended to treat confirmed cases as an unbiased measure of transmission intensity; rather, confirmed burden was used as an indicator of documented caseload and immediate response demand. The conceptual separation between epidemiological-operational and purely operational domains helps distinguish observed disease pressure from implementation constraints while retaining both within a single prioritization framework.

In a sensitivity analysis excluding confirmed burden and retaining the original absolute classification thresholds, all 13 confirmed affected health zones retained their original priority tier, indicating that the higher-priority classification of Rwampara, Mongbwalu, and Bunia was supported by convergence across multiple domains rather than by confirmed burden alone. Further validation using field-level data could assess whether differential domain weighting, rather than the equal weighting applied here, improves operational performance.

The dual fatality pathway was intended to accommodate changes in data maturity across outbreak phases. In the early phase examined here, suspected cases and deaths were more consistently disaggregated by health zone than confirmed outcomes, reflecting evolving testing capacity and outcome attribution: confirmed cases were reported by health zone at the 27 May data lock, but confirmed deaths were available only at the national level. The suspected-fatality pathway therefore supported early operational prioritization, while the confirmed-CFR pathway remains applicable when health-zone-level confirmed outcomes become available in later outbreak phases. Because the framework relied on public reporting, low scores could reflect either a genuinely limited operational signal or incomplete documentation; priority classifications were therefore reported alongside data classification confidence, particularly for newly affected or peripheral health zones.

### Comparison with existing evidence

These findings are consistent with early independent modelling that suggested substantial under-detection during the first phase of the outbreak [7]. This study did not replicate those models, but it provides operational evidence supporting the plausibility of under-detection, including the 20-day detection gap, early community deaths, HCW clusters, sample transport and testing bottlenecks, and laboratory backlog. The confirmed CFR by 27 May was lower than historical BVD estimates, but this should be interpreted cautiously because both confirmed cases and confirmed deaths were affected by testing backlog, incomplete ascertainment, and ongoing data harmonization. Historical evidence from the 2007 Uganda Bundibugyo outbreak and subsequent clinical analyses provides important context for the expected severity, diagnostic uncertainty, and early non-specific presentation of this virus [1,2].

### Strengths and limitations

This study has several strengths. It provides a timely, structured analysis of a rapidly evolving outbreak using publicly available data from national, regional, and international sources. It triangulates epidemiological, laboratory, contact-tracing, HCW exposure, security, and cross-border information, rather than relying only on case counts. It also proposes a transparent health-zone operational prioritization framework that can be updated as new public data become available.

The main limitation is the provisional nature of public outbreak data. The analysis relied on aggregated data that were revised during the response and cannot estimate individual-level risk factors, transmission chains, or clinical predictors. Some indicators were incomplete or unavailable for certain health zones, and public data may underestimate community deaths, unsafe burials, informal cross-border movements, security incidents, and unreported HCW exposures. The prioritization framework is not validated and should be interpreted as a transparent summarization tool rather than a predictive score. Because its numerical cut-points were empirically calibrated to the distribution observed during this early outbreak phase, recalibration is required before application to another epidemic phase, geographic setting, or outbreak. Finally, the exploratory growth estimate was constrained by a short time series and substantial laboratory backlog, while all reported indicators and health-zone priorities represent an early snapshot that may have changed after the data-lock dates.

## Conclusions

This early-phase operational snapshot indicates that the public picture available through the defined data-lock dates reflected both transmission and operational visibility, including how rapidly early signals were detected, verified, escalated, and linked to laboratory confirmation. In mobile, insecure, and hard-to-access communities, community-based and event-based surveillance should serve as the first operational line of defence. Unusual deaths, healthcare-worker clusters, rumours of severe illness, and unexplained febrile deaths should prompt rapid verification, specimen collection, alert escalation, and immediate IPC reinforcement before species-level confirmation is available.

The early findings support several operational implications. Laboratory response should prioritize rapid sample transport, surge testing capacity, and transparent reporting of samples pending testing, because backlog can delay outbreak visibility and distort confirmed case trends. IPC readiness should extend beyond the initial epicentre to referral hospitals, private facilities, border health posts, and urban receiving centres. Contact-tracing teams require surge capacity and clear procedures for addressing zero-contact or missing-contact reporting in confirmed affected zones. Risk communication and community engagement should be treated as core response functions because mistrust, misinformation, and insecurity can disrupt care, surveillance, contact follow-up, and safe isolation. Overall, these early findings support a response model centred on early signal detection, rapid laboratory confirmation, decentralized IPC readiness, strong contact tracing, trust-centred engagement, and transparent prioritization of health zones where multiple operational risks converge. These priorities should be reassessed as later outbreak data and more complete field information become available.

## Supporting information

S1 Checklist Completed STROBE checklist, reproduced under the Creative Commons Attribution 4.0 International (CC BY 4.0) licence [18]. (PDF)

S1 Table Health zone operational prioritization scoring framework for confirmed affected health zones. (PDF)

S2 Table Sensitivity analysis excluding the confirmed-burden domain from the health-zone operational prioritization framework. (PDF)

S1 Data Extracted analysis workbook containing the source data and derived indicators used in the study. (XLSX)
