# Supplementary material for: Operational epidemiology of the early phase of the 2026 Bundibugyo virus disease outbreak in the Democratic Republic of the Congo and Uganda
Source: PLOS Glob Public Health. 2026 Aug 27;6(8):e0006680. doi: 10.1371/journal.pgph.0006680 (PMC13521391; doi:10.1371/journal.pgph.0006680)
Supplement: Supplementary file 1 [file pgph.0006680.s001.pdf]

S1 Checklist. STROBE checklist

Operational epidemiology of the early phase of the 2026 Bundibugyo virus disease outbreak in the Democratic Republic of the Congo and Uganda

Checklist completed using the STROBE Statement for observational studies. The study is a retrospective public-data operational epidemiology analysis using aggregated, non-identifiable outbreak data. Participant-level items are therefore marked as not applicable where relevant, while source selection, missingness, sensitivity analyses, and health-zone classification are reported at the aggregate level.

STROBE item	Recommendation	Manuscript location / response
**Title and abstract**		
**1(a)**	Indicate the study design with a commonly used term in the title or abstract.	Title and Abstract. The title identifies the investigation as operational epidemiology of the early outbreak phase; the Abstract states that this was a retrospective public-data operational epidemiology study.
**1(b)**	Provide an informative and balanced summary of what was done and what was found.	Abstract. Summarizes the public data sources, timeline reconstruction, epidemiological and operational findings, health-zone prioritization, data classification confidence, and the sensitivity analysis excluding confirmed burden.
**Introduction**		
**2**	Explain the scientific background and rationale for the investigation being reported.	Introduction. Describes BVD, the 2026 DRC-Uganda outbreak, diagnostic uncertainty, cross-border mobility, insecurity, health-system constraints, and the central rationale that observed case trends reflect both transmission and operational visibility.
**3**	State specific objectives, including any prespecified hypotheses.	End of Introduction. States the objective to reconstruct the early trajectory and operational bottlenecks and to generate a transparent decision-support framework. No prespecified individual-level causal hypothesis was tested.
**Methods**		
**4**	Present key elements of study design early in the paper.	Methods, Study design. Presents the retrospective public-data operational epidemiology design, combining aggregate quantitative analysis with structured documentary analysis.
**5**	Describe the setting, locations, relevant dates, including periods of recruitment, exposure, follow-up, and data collection.	Methods, Study setting and period. Specifies affected DRC health zones in Ituri, North Kivu, and South Kivu; Uganda for imported and healthcare-linked cases; South Sudan as preparedness context; the primary period of 24 April-27 May 2026; and Uganda/regional triangulation through 29 May.
**6(a)**	Give eligibility criteria and sources and methods of selection of participants.	Methods, Data sources; Unit of analysis; Two-denominator framework. Not participant-based. Defines eligible public aggregate sources, health-zone/reporting-date units, confirmed affected health zones, and operationally monitored zones.
**6(b)**	For matched studies, give matching criteria and number of exposed and unexposed.	Not applicable. The study did not use matched participants, exposed/unexposed groups, or individual-level comparison groups.
**7**	Clearly define all outcomes, exposures, predictors, potential confounders, and effect modifiers. Give diagnostic criteria if applicable.	Methods, Variables and indicators; Detection gap analysis; Laboratory, contact tracing, and IPC indicators; Operational prioritization framework. Defines epidemiological and response variables, derived indicators, operational

STROBE item	Recommendation	Manuscript location / response
		detection gaps, the eight prioritization domains, the dual fatality pathways, priority tiers, and data classification confidence.
**8**	For each variable of interest, give sources of data and details of methods of assessment. Describe comparability of assessment methods if there is more than one group.	Methods, Data sources; Source hierarchy and reconciliation; Variables and indicators. Describes official situation reports, ministry statements, WHO and Africa CDC updates, selected partner statements, source prioritization, reconciliation of conflicting figures, and treatment of explicit zero, missing, and NA values.
**9**	Describe any efforts to address potential sources of bias.	Methods, Source hierarchy and reconciliation; Handling of unassigned confirmed cases; Operational prioritization framework; Sensitivity analyses. Addresses provisional public data, source discrepancies, incomplete reporting, unassigned cases, data classification confidence, alternative detection-gap definitions, and reclassification after excluding confirmed burden.
**10**	Explain how the study size was arrived at.	Methods, Study setting and period; Data sources; Two-denominator framework. The study included all eligible public aggregate outbreak records through the defined data locks, including 13 confirmed affected DRC health zones; no sample-size calculation was applicable.
**11**	Explain how quantitative variables were handled in the analyses. If applicable, describe which groupings were chosen and why.	Methods, Variables and indicators; Operational prioritization framework; Growth analysis; S1 Table. Describes cumulative counts, ratios, proportions, contacts per case, detection intervals, the exploratory log-linear estimate, 0-2 domain scores, empirically calibrated rounded cut-points, fixed priority thresholds, and data-completeness confidence categories.
**12(a)**	Describe all statistical methods, including those used to control for confounding.	Methods, Detection gap analysis; Epidemiological trajectory and geographic spread; Growth analysis; Sensitivity analyses. Describes calendar-day intervals, descriptive ratios and proportions, an exploratory log-linear analysis, and prespecified sensitivity checks. Confounding adjustment was not applicable because no individual-level causal modelling was performed.
**12(b)**	Describe any methods used to examine subgroups and interactions.	Methods and Results. Presents descriptive stratification by country, province, health zone, confirmed versus operationally monitored status, and HCW or healthcare-linked exposure. No formal interaction analysis was performed.
**12(c)**	Explain how missing data were addressed.	Methods, Source hierarchy and reconciliation; Handling of unassigned confirmed cases; Operational prioritization framework. Missing, unreported, not-determined, or insufficiently disaggregated data were treated as unavailable rather than zero. Explicit zero values remained assessable. Six unassigned confirmed cases were retained in national totals but excluded from health-zone analyses; classification confidence reflects domain-level data completeness.
**12(d)**	If applicable, describe analytical methods taking account of sampling strategy.	Not applicable. The study analysed public aggregate outbreak records rather than a probability sample.

STROBE item	Recommendation	Manuscript location / response
**12(e)**	Describe any sensitivity analyses.	Methods, Sensitivity analyses; Results, Sensitivity analysis; S2 Table. Includes confirmed-only analysis, alternative detection-gap start dates with a fixed 14 May laboratory-detection endpoint, exclusion of unassigned cases, assessment of the 23 May suspected-death discrepancy, and exclusion of confirmed burden while retaining the original absolute tier thresholds.
**Results**		
**13(a)**	Report numbers of individuals at each stage of the study.	Results, Early detection and governance timeline; Epidemiological trajectory and geographic spread; Operational response indicators. Not participant-based; reports country-, province-, health-zone-, case-, contact-, sample-, alert-, and healthcare-worker counts at relevant reporting stages.
**13(b)**	Give reasons for non-participation at each stage.	Not applicable. There were no participants or recruitment process. Missing, unassigned, and non-assessable public data are described in Methods and Results.
**13(c)**	Consider use of a flow diagram.	Not applicable. No participant flow diagram was required. Figures 1-3 summarize the detection and governance timeline, outbreak progression and geographic spread, and health-zone operational prioritization.
**14(a)**	Give characteristics of study participants and information on exposures and potential confounders.	Results, Geographic expansion; Uganda case progression; Health-worker and healthcare-linked IPC risk; Operational response indicators. Not participant-based; reports aggregate characteristics by country, province, health zone, exposure category, contacts, laboratory status, and operational indicators.
**14(b)**	Indicate number of participants with missing data for each variable of interest.	Methods, Source hierarchy and reconciliation; Operational prioritization framework; Results, Contact tracing and contact burden; Operational risk prioritization. Missingness is reported explicitly: six confirmed cases were geographically unassigned; contact-network size was NA for Kalunguta, Oicha, and Miti-Murhesa; classifications had high data confidence for 10 zones and moderate confidence for three.
**14(c)**	Summarize follow-up time where applicable.	Not applicable to individual participant follow-up. The outbreak reporting period and surveillance/contact-follow-up indicators are summarized by reporting date.
**15**	Report numbers of outcome events or summary measures.	Results, DRC confirmed and suspected case progression; Uganda case progression; Operational response indicators; Operational risk prioritization. Reports cases, deaths, suspected outcomes, contacts, alerts, laboratory testing and backlog, HCW cases, priority scores, tiers, and data classification confidence.
**16(a)**	Give unadjusted estimates and, if applicable, confounder-adjusted estimates and their precision.	Results. Reports descriptive unadjusted estimates, including confirmed CFR, suspected fatality proportion, contact-to-case ratios, laboratory positivity and backlog, the exploratory growth estimate, domain totals, priority tiers, and data classification confidence. Adjusted estimates were not applicable.
**16(b)**	Report category boundaries when continuous variables were categorized.	Methods, Operational prioritization framework; S1 Table. Reports boundaries for 0-2 domain scores, suspected-fatality and confirmed-CFR pathways,

STROBE item	Recommendation	Manuscript location / response
		confirmed-CFR denominator and cut-points, total scores of 0-16, priority tiers (0-4, 5-8, 9-16), and high/moderate/low data classification confidence.
**16(c)**	If relevant, consider translating estimates of relative risk into absolute risk for a meaningful time period.	Not applicable. The study did not estimate relative risks; it reports absolute counts, proportions, elapsed intervals, and operational indicators by reporting date.
**17**	Report other analyses done, including subgroup analyses and sensitivity analyses.	Results, Sensitivity analysis; Operational risk prioritization; Uganda case progression; Health-worker and healthcare-linked IPC risk; S2 Table. Reports country- and health-zone summaries, strict and broader healthcare-linked exposure indicators, and sensitivity analyses. Excluding confirmed burden changed no health-zone priority tier.
**Discussion**		
**18**	Summarize key results with reference to study objectives.	Discussion, Principal findings. Summarizes detection delay, geographic expansion, laboratory and contact-tracing pressure, HCW exposure, trust and security constraints, and the higher-priority health zones.
**19**	Discuss limitations, taking into account sources of potential bias or imprecision.	Discussion, Strengths and limitations. Addresses provisional and revised public data, aggregate-level inference, missing indicators, under-reporting, unmeasured events, equal domain weighting, the non-validated prioritization framework, and the short time series with laboratory backlog.
**20**	Give a cautious overall interpretation of results considering objectives, limitations, multiplicity of analyses, and evidence from similar studies.	Discussion, Interpreting confirmed case trends and detection delay; Operational prioritization and public health decision-making; Comparison with existing evidence; Conclusions. Interprets confirmed counts as products of transmission and operational visibility, considers the burden-exclusion sensitivity result, and situates findings against early modelling, historical BVD evidence, and IPC/community-surveillance literature.
**21**	Discuss generalisability of the study results.	Discussion, Strengths and limitations; Conclusions; S1 Table. Generalisability is framed for operational learning in mobile, insecure, hard-to-access, cross-border outbreak settings. The scoring thresholds are outbreak- and phase-specific and require recalibration before use in other settings.
**Other information**		
**22**	Give the source of funding and the role of the funders.	Funding. States that the authors received no specific funding for the work; therefore, no funder had a role in study design, analysis, interpretation, or reporting.

Abbreviations: BVD, Bundibugyo virus disease; CFR, case fatality ratio; COUSP/INSP, Centre des Opérations d’Urgence de Santé Publique / Institut National de Santé Publique; DRC, Democratic Republic of the Congo; HCW, healthcare worker; IPC, infection prevention and control; NA, not assessable; STROBE, Strengthening the Reporting of Observational Studies in Epidemiology; WHO, World Health Organization.
