# Supplementary material for: Operational epidemiology of the early phase of the 2026 Bundibugyo virus disease outbreak in the Democratic Republic of the Congo and Uganda
Source: PLOS Glob Public Health. 2026 Aug 27;6(8):e0006680. doi: 10.1371/journal.pgph.0006680 (PMC13521391; doi:10.1371/journal.pgph.0006680)
Supplement: Supplementary file 2 [file pgph.0006680.s002.pdf]

S1 Table. Health zone operational prioritization scoring framework for confirmed affected health zones

This supplementary table presents the framework scoring rubric used to classify confirmed affected health zones into lower-, moderate-, and higher-priority tiers at the 27 May 2026 data lock. The framework comprises three epidemiological-operational domains and five purely operational domains. Each assessable domain is scored from 0 to 2, giving a maximum total score of 16. The framework is descriptive and intended to support transparent operational prioritization using publicly available outbreak information. It was not designed or validated as a predictive or forecasting model.

Domain group	Domain	Score 0	Score 1	Score 2	Application notes
**EPIDEMIOLOGICAL-OPERATIONAL DOMAINS**	**Confirmed burden**	No confirmed case	1–9 confirmed cases	10 or more confirmed cases	*Confirmed burden represents documented caseload and immediate response demand. Because the framework is applied only to confirmed affected health zones, all zones included in the primary analysis score at least 1 in this domain. Confirmed burden is influenced by case recognition, testing capacity, laboratory throughput and reporting completeness and should not be interpreted as an unbiased measure of underlying transmission.*
	**Suspected mortality burden**	Zero suspected deaths explicitly reported	1–19 suspected deaths	20 or more suspected deaths	*This domain uses the absolute number of suspected deaths, not a proportion. Missing, unreported, not determined or insufficiently disaggregated suspected-death data are classified as NA and must not be scored as 0.*
	**Fatality proportion, suspected or confirmed**	Suspected fatality proportion below 10%; or explicitly reported zero suspected cases and zero suspected deaths, indicating no suspected-fatality signal; or confirmed CFR below 20% where at least five confirmed cases and zone-level confirmed deaths are available.	Suspected fatality proportion from 10% to below 20%; or confirmed CFR from 20% to below 40% where at least five confirmed cases and zone-level confirmed deaths are available.	Suspected fatality proportion of 20% or higher; or confirmed CFR of 40% or higher where at least five confirmed cases and zone-level confirmed deaths are available.	*The suspected-fatality pathway is calculated as suspected deaths divided by suspected cases where both values are explicitly available. An explicitly reported combination of zero suspected cases and zero suspected deaths is treated as an assessable absence of a suspected-fatality signal and receives score 0; no 0/0 proportion is calculated.*<br>*The confirmed-CFR pathway is calculated as confirmed deaths divided by confirmed cases and is assessable only when zone-level confirmed deaths are available and at least five confirmed cases are reported. The minimum confirmed-case denominator reduces instability from very small case counts. Where both pathways are assessable, the higher score is retained. Where neither pathway is assessable, the domain is recorded as NA.*<br>*At the 27 May 2026 data lock, confirmed cases were reported by health zone but confirmed deaths were not disaggregated by health zone. Consequently, the confirmed-CFR pathway did not contribute to the present health-zone scores; the suspected-fatality pathway was applicable during this early phase when suspected cases and deaths were more consistently reported at health-zone level. The confirmed-CFR pathway is retained as a phase-adaptive pathway that may become more applicable as testing and confirmed-outcome reporting mature; it is not a limitation of the framework.*

Domain group	Domain	Score 0	Score 1	Score 2	Application notes
PURELY OPERATIONAL DOMAINS	**Contact network size**	Zero listed contacts explicitly reported	1–199 listed contacts	200 or more listed contacts	*This domain reflects contact-tracing workload. Missing, unreported or not determined contact counts are recorded as NA and must not be treated as zero. Contact network size is scored independently from contact coverage gap.*
	**Contact coverage gap**	Contact information available with no evident listing or reporting gap	Missing, incomplete or very limited contact information despite confirmed cases or suspected deaths	Confirmed case or suspected death with explicitly zero listed contacts, or a documented major mismatch suggesting a substantial listing gap	*This domain assesses whether available contact information appears adequate relative to documented epidemiological activity. Missing contact counts may therefore make contact network size non-assessable while simultaneously supporting a contact coverage-gap score of 1. Score 2 is reserved for explicitly reported zero contacts despite confirmed activity or for a clearly documented major mismatch. In the present analysis, Kalunguta, Oicha, and Miti-Murhesa were therefore classified as NA for contact-network size and scored 1 for contact-coverage gap because their contact counts were missing or not determined, whereas Aru was scored 0 for contact-network size and 2 for contact-coverage gap because zero contacts were explicitly reported despite confirmed activity.*
	**IPC or health-worker risk**	No or low documented IPC or health-worker signal	Documented IPC gap, healthcare-facility exposure or isolated health-worker infection	Health-worker cluster, health-worker death, major IPC failure or suspected healthcare-facility amplification	*Scores are assigned from documented public evidence. Score 0 should be used only when available sources provide sufficient domain-relevant information to support the absence of a qualifying signal. Complete absence of IPC or health-worker information is recorded as NA rather than score 0.*
	**Security or community-trust risk**	No or low documented security or community-trust signal	Rumours, mistrust, insecurity or resistance affecting response risk	Direct response disruption, attack, arson, major resistance or severe access constraint	*Security and community-trust signals are scored only when supported by official or selected partner documentation. Score 0 requires sufficient domain-relevant information; absence of any information is recorded as NA.*
	**Mobility or cross-border relevance**	No or low documented mobility relevance	Local or inter-health-zone mobility concern	Major urban hub, mining corridor, border corridor, international movement or cross-border relevance	*Mobility may increase strategic importance independently of documented case burden. Score 0 requires sufficient contextual information to support low mobility relevance; absence of mobility information is recorded as NA.*

Not assessable (NA): NA is not a domain score and is distinct from score 0. A domain is recorded as NA when the information required to assign a score is missing, unreported, not determined, insufficiently disaggregated or otherwise non-assessable. NA contributes no points to the observed total, but the score remains reported against the common maximum of 16. Incomplete information is reflected through data classification confidence and may result in underestimation of operational priority.

Total score	Priority tier
**0–4**	Lower priority
**5–8**	Moderate priority
**9–16**	Higher priority

Equal domain weighting was retained to support transparency and reproducibility and because no validated evidence was available to justify differential weights. Equal weighting should not be interpreted as evidence that all domains have equivalent causal or predictive importance.

Assessable domains	Data classification confidence
**8 of 8**	High
**5–7 of 8**	Moderate
**Fewer than 5 of 8**	Low

A domain is counted as assessable when sufficient information is available to assign a score of 0, 1 or 2. Explicitly reported zero values and clearly documented absence of a qualifying signal count as available information. Missing, unreported, not determined, insufficiently disaggregated or otherwise non-assessable information does not count as available. Data classification confidence reflects the completeness of the publicly available evidence supporting the classification. It does not represent statistical confidence, predictive validity or confirmation of the accuracy of the underlying data.

Threshold basis: Numerical cut-points used in the primary analysis were empirically derived from the distribution of health-zone values available at the 27 May 2026 data lock and were rounded to support transparent operational interpretation. They were selected to distinguish limited, intermediate and upper-range documented signals within this early-outbreak dataset. The cut-points are not universal Ebola response standards or validated prognostic thresholds. Recalibration is required before applying the framework to another epidemic phase, geographic setting or outbreak. The confirmed-CFR pathway was retained as a phase-adaptive alternative but did not affect the present analysis because health-zone-level confirmed deaths were unavailable at the data lock.

The epidemiological-operational domains describe documented disease and outcome pressure generated jointly by underlying transmission and operational visibility, including case recognition, testing, laboratory throughput and reporting completeness. The purely operational domains describe response workload, implementation gaps and potential amplification conditions. The composite score supports transparent operational prioritization and is not an estimate of underlying transmission intensity.

Scores used public information available at the defined analysis lock. Absence of a publicly documented signal does not imply absence of operational risk, and sparse reporting may underestimate priority, particularly in newly affected, insecure or peripheral health zones. Scores should therefore be interpreted jointly with domain-level evidence and data classification confidence.
