# Supplementary material for: Operational epidemiology of the early phase of the 2026 Bundibugyo virus disease outbreak in the Democratic Republic of the Congo and Uganda
Source: PLOS Glob Public Health. 2026 Aug 27;6(8):e0006680. doi: 10.1371/journal.pgph.0006680 (PMC13521391; doi:10.1371/journal.pgph.0006680)
Supplement: Supplementary file 3 [file pgph.0006680.s003.pdf]

S2 Table. Sensitivity analysis excluding the confirmed-burden domain from the health-zone operational prioritization framework

This sensitivity analysis assessed whether inclusion of the confirmed-burden domain materially influenced health-zone priority classification. The confirmed-burden score was removed from each health zone's final framework total, while the scores for the remaining seven domains were retained unchanged.

Province	Health zone	Confirmed-burden score	Primary framework total score	Primary priority tier	Sensitivity score excluding confirmed burden	Sensitivity priority tier	Tier changed
Ituri	**Rwampara**	2	**14/16**	**Higher**	12/14	**Higher**	No
	**Mongbwalu**	2	**12/16**	**Higher**	10/14	**Higher**	No
	**Bunia**	2	**11/16**	**Higher**	9/14	**Higher**	No
	**Nyankunde**	2	**8/16**	**Moderate**	6/14	**Moderate**	No
	**Nizi**	1	**7/16**	**Moderate**	6/14	**Moderate**	No
	**Aru**	1	**7/16**	**Moderate**	6/14	**Moderate**	No
	**Kilo**	1	**6/16**	**Moderate**	5/14	**Moderate**	No
North Kivu	**Goma**	1	**4/16**	**Lower**	3/14	**Lower**	No
	**Butembo**	1	**3/16**	**Lower**	2/14	**Lower**	No
	**Katwa**	1	**3/16**	**Lower**	2/14	**Lower**	No
	**Kalunguta**	1	**3/16**	**Lower**	2/14	**Lower**	No
	**Oicha**	1	**3/16**	**Lower**	2/14	**Lower**	No
South Kivu	**Miti-Murhesa**	1	**3/16**	**Lower**	2/14	**Lower**	No

Note: Sensitivity classification: The original absolute priority cut-points were retained without recalibration: lower priority, 0–4; moderate priority, 5–8; and higher priority, ≥9. Because the confirmed-burden domain was excluded, the maximum possible sensitivity score was 14 rather than 16.

Interpretation: Excluding confirmed burden reduced the numerical score of every health zone but did not change the priority tier of any of the 13 health zones. Rwampara, Mongbwalu and Bunia remained higher priority, indicating that their classifications were supported by multiple epidemiological-operational and purely operational domains rather than confirmed burden alone.

This sensitivity analysis assesses the dependence of the classifications on the confirmed-burden domain. It does not validate the framework, establish predictive performance or imply that confirmed case counts are unaffected by testing capacity, laboratory throughput or other components of operational visibility.
