# Supplementary material for: Operational epidemiology of the early phase of the 2026 Bundibugyo virus disease outbreak in the Democratic Republic of the Congo and Uganda
Source: PLOS Glob Public Health. 2026 Aug 27;6(8):e0006680. doi: 10.1371/journal.pgph.0006680 (PMC13521391; doi:10.1371/journal.pgph.0006680)
Supplement: Supplementary file 4 [file pgph.0006680.s006.pdf]

Response to the Academic Editor and Reviewers

Manuscript ID: PGPH-D-26-02057

Operational epidemiology of the early phase of the 2026 Bundibugyo virus disease outbreak in the Democratic Republic of the Congo and Uganda

PLOS Global Public Health

Dear Dr Fernandez and Reviewers,

We thank the Academic Editor and both reviewers for their careful assessment and constructive recommendations. We have revised the manuscript substantially to clarify its central scientific argument, strengthen the health zone operational prioritization framework, improve reproducibility, and enhance the presentation of all figures. The revised manuscript now explicitly frames reported outbreak indicators as products of both underlying transmission and operational visibility; separates epidemiological-operational from purely operational domains; introduces data classification confidence; applies a revised phase-adaptive fatality domain; and includes a confirmed-burden-excluded sensitivity analysis. Figures 1 and 3 were comprehensively redesigned, and the specific requested corrections were made to Figure 2. S1 Table now provides the complete framework scoring rubric and threshold rationale, while S2 Table reports the burden-exclusion sensitivity analysis.

All manuscript changes are highlighted in the marked-up version. Page and line references below refer to the revised marked-up manuscript.

Summary of principal revisions

Area	Revision implemented
**Scientific framing**	The Introduction, Abstract, Discussion, and Conclusion now state that confirmed-case curves, CFRs, and apparent growth reflect both transmission and operational visibility.
**Prioritization framework**	The eight domains are grouped into three epidemiological-operational and five purely operational domains; confirmed burden is explicitly interpreted as documented caseload and response demand, not as an unbiased transmission measure.
**Robustness analysis**	A seven-domain sensitivity analysis excluding confirmed burden retained the original absolute cut-points; none of the 13 health zones changed priority tier.
**Data completeness**	Data classification confidence is now reported as high, moderate, or low; 10 zones had high confidence and three had moderate confidence.
**Figures**	Figure 1 was comprehensively redesigned; Figure 2 received the two requested terminology corrections; Figure 3 now displays framework scores, domain grouping, NA values, and classification confidence.
**Supporting information**	S1 Table contains the full framework rubric, missing-versus-zero rules, equal-weighting rationale, empirical threshold basis, and recalibration requirement. S2 Table presents the confirmed-burden-excluded sensitivity analysis.

Academic Editor

Journal requirement 1: Ethics Statement placement

> ***Comment:*** *Please ensure that your Ethics Statement is available in its entirety at the beginning of your Methods section, under a subheading 'Ethics Statement'.*

Response: We have moved the complete Ethics Statement to the beginning of the Materials and methods section under the required subheading “Ethics statement.” No substantive ethical information was removed.

Location of changes: Materials and methods, p. 6, lines 115–123.

Reviewer 1

Major comment 1: State the unifying scientific argument earlier

> ***Comment:*** *The unifying hypothesis appears to be that, during the early outbreak phase, observed confirmed cases, deaths, CFR, and the growth curve are jointly produced by true transmission and operational visibility. Detection delay, laboratory throughput, contact-listing completeness, and security access shape what becomes visible; therefore, confirmed-case curves should not be read as transmission curves. Recommends stating this more succinctly earlier in the paper.*

Response: We agree and have made this the explicit conceptual premise of the paper. A new paragraph near the end of the Introduction now states that publicly observed confirmed case counts, CFRs, and growth trajectories are joint products of underlying transmission and the operational systems that detect, test, and report cases. The Abstract was also strengthened to summarize this interpretation, and the Discussion now reiterates that confirmed-case trends cannot be interpreted as transmission trends in isolation.

Location of changes: Abstract, pp. 2–3, lines 24–47; Introduction, p. 5, lines 94–114; Discussion, section “Interpreting confirmed case trends and detection delay,” and Conclusion.

Key revised text: *“In the early phase of high-consequence outbreaks in fragile settings, what becomes visible through public data, including confirmed case counts, case fatality ratios, and growth trajectories, is a joint product of true transmission dynamics and operational factors such as detection capacity, laboratory throughput, reporting completeness, and access. This study treats confirmed epidemiological indicators not as direct measures of transmission but as imperfect observational proxies shaped by the operational system that generates them.”*

Major comment 2: Circularity, weighting, and whether the framework should remain scored

> ***Comment:*** *The framework integrates epidemiological and operational elements, but “high burden of cases” may itself reflect operational challenges, making the ranking partly circular. The reviewer asks whether the framework would work better as a structured summary rather than a scored instrument, given that the domain weighting has not been validated.*

Response: We agree that confirmed burden requires explicit qualification and that the original presentation did not sufficiently separate epidemiological pressure from operational constraints. We retained the scored framework because it is intended as a transparent operational prioritization instrument rather than a prediction model, but we substantially revised its conceptual structure, implementation, and validation claims.

Location of changes: Methods, section “Health zone Operational prioritization framework,” pp. 10–13, lines 206–274; Sensitivity analyses, pp. 13–14, lines 275–297; Results, pp. 22–23, lines 478–500; Discussion, pp. 27–28, lines approximately 590–612; revised Figure 3; S1 Table; and S2 Table.

- The eight domains are now divided into three epidemiological-operational domains (confirmed burden, suspected mortality burden, and fatality proportion based on suspected or confirmed outcomes) and five purely operational domains (contact network size, contact coverage gap, IPC/health-worker risk, security/community-trust risk, and mobility/cross-border relevance).
- Confirmed burden is explicitly retained as an indicator of documented caseload and immediate response demand, while the manuscript states that it is influenced by detection, testing, laboratory throughput, and reporting completeness and is not an unbiased measure of transmission intensity.

- Equal weighting is justified as a transparent and reproducible choice in the absence of validated evidence for differential weights; the manuscript explicitly states that equal weighting does not imply equivalent causal or predictive importance.
- We conducted an additional sensitivity analysis excluding confirmed burden while retaining the original absolute priority cut-points. None of the 13 health zones changed priority tier; Rwampara, Mongbwalu, and Bunia remained higher priority. The full results are provided in S2 Table.
- We introduced data classification confidence based on domain assessability. Ten health zones had high confidence and three had moderate confidence; no classification had low confidence.
- The framework fatality domain now uses a phase-adaptive suspected-fatality/confirmed-CFR pathway, and NA is distinguished from an explicitly supported score of zero.
- S1 Table now provides the complete rubric, threshold basis, missing-versus-zero rules, equal-weighting rationale, and requirement for recalibration in other epidemic phases or settings.

Overall Response: These revisions preserve the practical value of a scored prioritization tool while directly addressing the concern that the final tiers might be driven by confirmed burden alone. The sensitivity analysis demonstrates that the classifications were supported by convergence across multiple domains; however, we continue to describe the framework as descriptive and unvalidated, not predictive.

Key revised text: *"The inclusion of confirmed burden as a domain requires careful interpretation because reported case counts reflect both underlying transmission and the operational system through which infections are detected, tested, and reported. Its inclusion was therefore not intended to treat confirmed cases as an unbiased measure of transmission intensity; rather, confirmed burden was used as an indicator of documented caseload and immediate response demand."*

Applicable minor comments from Reviewer 1

Minor comment 1: Clarify INSP and COUSP

> ***Comment:*** *The phrase "public health emergency operation centre (INSP/COUSP)" is ambiguous. Please provide the full official names and use the acronyms consistently.*

Response: We revised the institutional description to make clear that COUSP-RDC is DRC Centre des Opérations d'Urgence de Santé Publique within the Institut National de Santé Publique (INSP), and we use the acronyms consistently thereafter.

Location of changes: Data sources, p. 7, lines 138–150.

Key revised text: *"Primary DRC data were obtained from situation reports issued by the Centre des Opérations d'Urgence de Santé Publique de la République démocratique du Congo (COUSP-RDC), within the Institut National de Santé Publique (INSP)."*

Minor comment 2: Define detection gap

> ***Comment:*** *Please provide a brief definition of "detection gap" before presenting the calculated intervals.*

Response: We added a direct operational definition before listing the intervals.

Location of changes: Detection gap analysis, pp. 9–10, lines 183–205.

Key revised text: *"The primary outcome was the operational detection gap, defined as the elapsed time between successive milestones in outbreak recognition and response."*

Minor comment 3: Clarify recalculated intervals and fixed endpoint

> ***Comment:*** *The manuscript states that alternative detection-gap definitions were evaluated using symptom onset, community signal, and official notification dates, but it is unclear which intervals were recalculated or which endpoint remained fixed.*

Response: We clarified that first laboratory detection on 14 May remained the fixed endpoint, while the starting milestone was varied among symptom onset on 24 April, first community signal on 5 May, and official alert notification on 8 May. This yields three interval lengths to the same fixed endpoint.

Location of changes: Sensitivity analyses, pp. 13–14, lines 275–297.

Minor comment 4: Remove extra closing bracket

> ***Comment:*** *Please remove the extra closing bracket "]".*

Response: The extra closing bracket after the citation at the DRC declaration paragraph was removed.

Location of changes: Results, DRC confirmed and suspected case progression, p. 16, beginning around line 344.

Reviewer 2

Major comment 1a: Figure 1 timeline axis and date labels

> ***Comment:*** *Date formatting is inconsistent and some dates are difficult to read because dashed event stems run behind them. Please reformat the timeline axis to better illustrate the month and dates, considering the PATH example.*

Response: Figure 1 was comprehensively redesigned. The revised figure uses a continuous calendar-based month band for April and May 2026, consistent date formatting, separate epidemiological/operational and governance lanes, and date labels that are no longer crossed by event stems. The detection-gap panel is visually separated below the main timeline.

Location of changes: Revised Figure 1 and caption, pp. 15–16, lines approximately 329–343.

Major comment 1b: Figure 1 event stems and markers

> ***Comment:*** *For some events, including the DRC official declaration, it is unclear which event stem is attached. Branching lines may help.*

Response: We replaced ambiguous stems with explicit elbow and branching connectors. The events occurring on 15 May—species-level confirmation, Uganda imported-case confirmation, and official DRC declaration—now originate from the same 15 May anchor and branch to their respective labels. Other clustered governance events use staggered elbow connectors to preserve one-to-one linkage between event and date.

Location of changes: Revised Figure 1.

Major comment 1c: Figure 1 colour coding

> ***Comment:*** *The colour coding of event stems and markers is inconsistent. The official alert notification corresponds to Event C and should be dark brown, while events after 15 May need a clearer legend or a uniform colour.*

Response: We corrected the official alert notification to the Gap C colour and standardized the A–E gap colours throughout the figure. Events after the final detection-gap milestone that are not gap endpoints are displayed uniformly in neutral grey. The lower panel directly labels Gaps A–E and their elapsed durations, making the colour logic explicit without relying on ambiguous event colouring.

Location of changes: Revised Figure 1 and caption, pp. 15–16, lines approximately 329–343.

Minor comment 1: Spell out WASH

> ***Comment:*** *Line 114: Please spell out WASH (water, sanitation, and hygiene) at first use.*

Response: WASH is now spelled out at first use.

Location of changes: Study design, p. 6, lines 124–137.

Key revised text: *"IPC and water, sanitation, and hygiene (WASH)."*

Minor comment 2: Clarify INSP/COUSP

> ***Comment:*** *The phrase "public health emergency operation centre (INSP/COUSP)" is ambiguous; it is unclear whether INSP and COUSP refer to separate institutions or a single entity.*

Response: We clarified the institutional relationship and standardized the terminology throughout the manuscript.

Location of changes: Data sources, p. 7, lines 138–150.

Key revised text: *"Primary DRC data were obtained from situation reports issued by the Centre des Opérations d'Urgence de Santé Publique de la République démocratique du Congo (COUSP-RDC), within the Institut National de Santé Publique (INSP)."*

Minor comment 3: Define detection gap

> ***Comment:*** *Please provide a brief definition of "detection gap" before presenting the calculated intervals.*

Response: We added the requested definition.

Location of changes: Detection gap analysis, pp. 9–10, lines 183–205.

Key revised text: *"The primary outcome was the operational detection gap, defined as the elapsed time between successive milestones in outbreak recognition and response."*

Minor comment 4: Clarify alternative detection-gap endpoints

> ***Comment:*** *It is unclear which detection-gap intervals were recalculated and which endpoint or endpoints remained fixed.*

Response: We clarified that first laboratory detection on 14 May was held as the fixed endpoint while the starting point was varied across symptom onset, first community signal, and official alert notification.

Location of changes: Sensitivity analyses, pp. 13–14, lines 275–297.

Minor comment 5: Remove extra closing bracket

> ***Comment:*** *Line 288: Please remove the extra closed bracket "]".*

Response: The extra bracket was removed from the citation following the declaration data.

Location of changes: Results, DRC confirmed and suspected case progression, p. 16.

Minor comment 6: Figure 2 terminology

> ***Comment:*** *Please replace the forward slash in the y-axis title "Confirmed cases/deaths" to avoid confusion with division. Also change "Nord- and Sud-Kivu" in the figure legend to North and South Kivu for consistency.*

Response: We made both requested corrections without changing the underlying data or graphical scales. The Panel A y-axis now reads "Confirmed cases and deaths," and the province names are presented in English as "North Kivu" and "South Kivu." The caption was also standardized accordingly.

Location of changes: Revised Figure 2 and caption, p. 17.

Minor comment 7: Resolve tracked change near the citation

> ***Comment:*** *Line 486: There appears to be a track change that needs to be resolved immediately before the in-text citation.*

Response: The unresolved tracked-change artefact was resolved. We also conducted a document-wide review to ensure that no unintended revision marks or unresolved editorial artefacts remain in the clean manuscript.

Location of changes: Revised manuscript, document-wide; the affected Results passage was corrected.

Minor comment 8: Explain the scoring-threshold basis

> ***Comment:*** *Please clarify how thresholds such as ≥10 confirmed cases, ≥20 suspected deaths, and ≥200 contacts were selected. Were these based on expert consensus, operational guidance, the observed data distribution, or another rationale?*

Response: We now state explicitly that the numerical cut-points used in the primary analysis were empirically derived from the distribution of health-zone values available at the 27 May 2026 data lock and rounded to distinguish limited, intermediate, and upper-range documented signals while remaining operationally interpretable. We also clarify that these are not universal Ebola response standards or validated prognostic thresholds and require recalibration before use in another outbreak, epidemic phase, or geographic setting. The confirmed-CFR pathway thresholds are separately identified as pre-specified phase-adaptive thresholds that did not affect the present analysis because health-zone-level confirmed deaths were unavailable.

Location of changes: Health zone operational prioritization framework, p. 11-12, lines 229–274; revised S1 Table, “Threshold basis” note; Strengths and limitations.

Key revised text: *“Numerical thresholds for the domains applied in the primary analysis were empirically calibrated to the observed health-zone data distribution at the analysis lock, with rounded cut-points selected to distinguish limited, intermediate, and upper-range documented signals while remaining operationally interpretable.”*

Additional revisions made to strengthen the manuscript

- The Abstract now reports that excluding confirmed burden did not change any health-zone tier and that data classification confidence was high for 10 zones and moderate for three.
- Figure 3 was revised to display the two domain groups, framework scores, NA values distinct from score 0, total score, priority tier, and data classification confidence.
- The fatality domain now uses a phase-adaptive suspected-fatality/confirmed-CFR pathway. At the 27 May data lock, confirmed deaths were not disaggregated by health zone, so the suspected-fatality pathway was the applicable pathway; this reflects early-phase data maturity rather than a limitation of the framework design.
- Missing or not-determined contact-network data are now classified as NA rather than zero. Contact coverage gap is scored separately, allowing missing contact data to indicate a reporting/listing gap without being misrepresented as a zero-sized contact network.
- The Data Availability statement now confirms that the extracted analysis workbook is provided as Supporting Information.
- The Supporting Information captions were updated to list the final S1 Table and S2 Table.

We appreciate the reviewers’ comments, which materially improved the conceptual clarity, reproducibility, and presentation of the manuscript. We hope that the revised manuscript and supporting information fully address the concerns raised.

Sincerely,

Tambe Elvis Akem
On behalf of the authors
