# Supplementary material for: Operational epidemiology of the early phase of the 2026 Bundibugyo virus disease outbreak in the Democratic Republic of the Congo and Uganda
Source: PLOS Glob Public Health. 2026 Aug 27;6(8):e0006680. doi: 10.1371/journal.pgph.0006680 (PMC13521391; doi:10.1371/journal.pgph.0006680)
Supplement: Supplementary file 5 [file pgph.0006680.s007.pdf]

Response to the Academic Editor

Manuscript ID: PGPH-D-26-02057R1

Title: Operational epidemiology of the early phase of the 2026 Bundibugyo virus disease outbreak in the Democratic Republic of the Congo and Uganda

Journal: PLOS Global Public Health

3 August 2026

Dear Dr Fernandez,

We thank you for the opportunity to revise the manuscript further. We appreciate your confirmation that the previous reviewer comments were addressed satisfactorily. We have now strengthened the temporal framing throughout the manuscript so that the findings are consistently presented as an early-phase operational snapshot anchored to the defined data-lock dates.

Additional Editor Comment

Comment

The authors have addressed the reviewer's comments in a satisfactory way. The only pending comment would be to strengthen the framing of the manuscript as an early-phase outbreak analysis tied to the stated data-lock dates. The manuscript should make clear that the case counts, operational indicators, health-zone prioritization, and interpretation reflect the public data available through the defined early analysis period, rather than the current or final status of the outbreak. Please also review the conclusion to ensure that recommendations are framed as implications from this early operational snapshot, not as conclusions about the full outbreak trajectory.

Response

We agree and have revised the manuscript accordingly. The temporal scope is now stated explicitly and consistently across the Abstract, Methods, Results, Discussion, Limitations, and Conclusions. The revised text clarifies that the primary DRC data lock was 27 May 2026, while Uganda and regional triangulation extended through 29 May 2026, and that the findings do not represent a current or final assessment of cumulative outbreak burden, geographic spread, response performance, or health-zone priority.

Revisions implemented

- **Abstract:** The data-lock dates are now stated explicitly, and the interpretation is framed as an early operational snapshot.
- **Study setting and period:** The Methods now explain that the data locks were retained to preserve the study's early-phase focus and were not updated to reflect subsequent outbreak evolution.
- **Health-zone prioritization:** The Methods state that all scores reflect public information available at the 27 May 2026 DRC data lock and are not current classifications beyond that date. The Results likewise tie the prioritization findings directly to the locked dataset.
- **Discussion and limitations:** The Principal findings section now opens by defining the analysis as an early snapshot rather than a current or complete assessment. The limitations clarify that reported indicators and health-zone priorities may have changed after the data-lock dates.

- **Conclusions:** The recommendations are now presented as operational implications arising from the early-phase snapshot, and the manuscript states that these priorities should be reassessed as later outbreak data and more complete field information become available.

Locations of changes: Abstract (pp. 2–3); Materials and methods, Study setting and period (p. 7) and Health-zone operational prioritization framework (p. 13); Results, Health-zone operational prioritization (pp. 22–23); Discussion, Principal findings (p. 25) and Strengths and limitations (p. 29); and Conclusions (p. 30).

No references were added, removed, or changed in response to this comment.

We appreciate this focused recommendation, which has improved the clarity of the manuscript's temporal scope and the interpretation of its operational implications.

Sincerely,

Tambe Elvis Akem
Corresponding Author
On behalf of the authors
