# Supplementary material for: Operational epidemiology of the early phase of the 2026 Bundibugyo virus disease outbreak in the Democratic Republic of the Congo and Uganda
Source: PLOS Glob Public Health. 2026 Aug 27;6(8):e0006680. doi: 10.1371/journal.pgph.0006680 (PMC13521391; doi:10.1371/journal.pgph.0006680)
Supplement: Supplementary file 6 [file pgph.0006680.s005.docx]

This manuscript reconstructs the early stages of the 2026 BVD outbreak in DRC and Uganda and presents the bottlenecks across key operational areas of the outbreak response. The study is valuable given the…By….., the findings may also….Strengths of the study include… My main concerns….The manuscript would also benefit from…Overall

Major comments:

1. *Clarification of Bayesian estimation framework (lines 167-170)*: Consider providing additional context for the Bayesian estimation procedures for readers less familiar with these methods. Briefly distinguish MCMC as the general framework from Gibbs sampling as the specific algorithm used and clarify why this approach was applied for parameter estimation. Please also relate the Gelman-Rubin statistic to the overall framework (e.g., “convergence of MCMC chains…”).
2. *Interpretation of R0 and its relationship to mean age at infection (lines 171-186)*: Please define R0 in epidemiological terms and provide additional interpretive context for the relationship R0 = L/A (e.g., younger mean age at infection corresponds to higher transmission intensity and larger R0).
3. *HIT and translation to vaccination coverage:* The HIT is calculated from R0 estimated based on natural infection. In this context, it’s reasonable that primary vaccine failure is not included. It would be helpful to note in the discussion, though, that this theoretical threshold does not directly translate to vaccination coverage targets, and that vaccine efficacy should be considered when determining the coverage needed to achieve herd immunity.
4. *Clarity and consistency of IgG concentrations and figures* (Figure 1B, S1A and S1B):
   a. Lines 216-222: There appears to be a lack of clarity regarding whether VZV IgG concentrations are reported on the original or log-transformed scale. The text describes “log-transformed VZV IgG antibody” but reports values in IU/mL that appear to correspond to back-transformed geometric mean concentrations, while Figure 1B appears to display log-transformed values (including negative means). Please clarify the scale used for both the statistical summaries and the figure to ensure consistency in interpretation. In addition, the numerical values reported in the text should be checked for consistency with those shown in Figure 1B, as there appear to be minor discrepancies. Also in line 220, “lowest” of what? E.g., Please correct to “lowest concentration of 0.04…”
   b. Lines 236-238: Please clarify if these values are averaged across years or referencing a specific year.
   c. Lines 242-244: Please describe the pattern (e.g., above the seropositivity threshold).
5. *Generalizability to other settings* (line 368): The study was conducted in a rural coastal setting, which as mentioned in the limitations section, may limit generalizability. Given that varicella transmission is strongly dependent on contact patterns and age structure, it would be helpful if the authors could discuss how FOI, R), or other estimates might differ in urban or higher density settings.

Minor comments:

- Lines 130 (141, etc.): The description of the primary data sources would benefit from greater clarity regarding the institutions involved. The phrase "public health emergency operation centre (INSP/COUSP)" is ambiguous, as it is unclear whether INSP and COUSP refer to separate institutions or a single entity. Please provide the full official names (in English and/or French, as appropriate) when first introduced and ensure the acronyms are used consistently throughout the manuscript.
- Lines 188-189: To improve clarity for PLOS GH readers not familiar with operational epidemiology, it may be helpful to provide a very brief definition of ‘detection gap’–something like “the elapsed time between successive milestones in outbreak recognition and response”–before jumping into the various intervals that were calculated.
- Lines 226-227: The manuscript states that alternative detection-gap definitions were evaluated using symptom onset, community signal, and official notification dates, but it is not clear which intervals were recalculated or which endpoint(s) remained fixed. A brief clarification would improve reproducibility.
- Line 288: Please remove the extra closed bracket “]”.
