# Supplementary material for: Operational epidemiology of the early phase of the 2026 Bundibugyo virus disease outbreak in the Democratic Republic of the Congo and Uganda
Source: PLOS Glob Public Health. 2026 Aug 27;6(8):e0006680. doi: 10.1371/journal.pgph.0006680 (PMC13521391; doi:10.1371/journal.pgph.0006680)
Supplement: Peer review history

# Peer review: Decision Letter 0

**DOI:** 10.1371/journal.pgph.0006680.r001

**Authors:** Maria del Pilar Fernandez

20 Jul 2026

PGPH-D-26-02057

Operational epidemiology of the early phase of the 2026 Bundibugyo virus disease outbreak in the Democratic Republic of the Congo and Uganda

PLOS Global Public Health

Dear Dr. Elvis Akem,

Thank you for submitting your manuscript to PLOS Global Public Health. After careful consideration, we feel that it has merit but does not fully meet PLOS Global Public Health’s publication criteria as it currently stands. Therefore, we invite you to submit a revised version of the manuscript that addresses the points raised during the review process.

Please submit your revised manuscript by Sep 18 2026 11:59PM. If you will need more time than this to complete your revisions, please reply to this message or contact the journal office at globalpubhealth@plos.org. When you're ready to submit your revision, log on to https://www.editorialmanager.com/pgph/ and select the 'Submissions Needing Revision' folder to locate your manuscript file.

Please include the following items when submitting your revised manuscript:

- A letter that responds to each point raised by the editor and reviewer(s). You should upload this letter as a separate file labeled 'Response to Reviewers'.
- A marked-up copy of your manuscript that highlights changes made to the original version. You should upload this as a separate file labeled 'Revised Manuscript with Track Changes'.
- An unmarked version of your revised paper without tracked changes. You should upload this as a separate file labeled 'Manuscript'.

Guidelines for resubmitting your figure files are available below the reviewer comments at the end of this letter.

As the corresponding author, your ORCID iD is verified in the submission system and will appear in the published article. PLOS supports the use of ORCID, and we encourage all coauthors to register for an ORCID iD and use it as well. Please encourage your coauthors to verify their ORCID iD within the submission system before final acceptance, as unverified ORCID iDs will not appear in the published article. *Only*  the individual author can complete the verification step; PLOS staff *cannot*  verify ORCID iDs on behalf of authors.

We look forward to receiving your revised manuscript.

Kind regards,

Maria del Pilar Fernandez

Academic Editor

PLOS Global Public Health

**Journal Requirements:**

1. Please ensure that your Ethics Statement is available in its entirety at the beginning of your Methods section, under a subheading 'Ethics Statement'.

If the reviewer comments include a recommendation to cite specific previously published works, please review and evaluate these publications to determine whether they are relevant and should be cited. There is no requirement to cite these works unless the editor has indicated otherwise.

**Additional Editor Comments (if provided):**

[Note: HTML markup is below. Please do not edit.]

Reviewers' comments:

Reviewer's Responses to Questions

**Comments to the Author**

1. Does this manuscript meet PLOS Global Public Health’s publication criteria?

Reviewer #1: Partly

Reviewer #2: Yes

**********

2. Has the statistical analysis been performed appropriately and rigorously?

Reviewer #1: Yes

Reviewer #2: Yes

**********

3. Have the authors made all data underlying the findings in their manuscript fully available (please refer to the Data Availability Statement at the start of the manuscript PDF file)?

The PLOS Data policy

Reviewer #1: Yes

Reviewer #2: Yes

**********

4. Is the manuscript presented in an intelligible fashion and written in standard English?

Reviewer #1: Yes

Reviewer #2: Yes

**********

**Reviewer #1:**  Akem and Oben share critical operational data about the current BDBV outbreak. If I am reading this correctly, I believe the unifying hypothesis (as they outline in discussion) is that in the early phase outbreak, what you observe (confirmed cases, deaths, CFR, the growth curve) is a joint product of true transmission and operational visibility. And hence, detection delay, lab throughput, contact-listing completeness, security access are all playing a role in what is visible. Therefore confirmed-case curves must not be read as transmission curves. If this is indeed the argument, I would suggest authors state this more succinctly earlier in the paper to help guide reader through the data presented later.

The paper's novel addition to publicly available data is a framework that integrates operational elements in addition epidemiological ones. Here, I am concerned that "high burden of cases" which itself is a reflection of some of the operational challenges is also included as a domain - which may make the framework ranking a bit circular. I wonder if framework would work better as a structured summary rather than a scored instrument, as there is no validation of the weighting for each domain etc.

**Reviewer #2:**  This manuscript reconstructs the early stages of the 2026 Bundibugyo virus disease (BVD) outbreak in DRC and Uganda and describes key operational bottlenecks encountered during the cross-country outbreak response. By synthesizing publicly available operational and epidemiological data into a chronological reconstruction and descriptive response analysis, the study provides a useful overview of the outbreak’s early trajectory and may help inform preparedness and response planning for future filovirus outbreaks. Strengths of the study include its timely focus, transparent use of publicly available data sources, and its integration of epidemiological and operational response measures. My main concern relates to the presentation of Figure 1, where improvements in visual clarity would substantially enhance communication of the findings. The manuscript would also benefit from several minor clarifications to improve transparency, reproducibility, and accessibility for a broader public health audience. Overall, I believe the study is a valuable contribution and is very well written, but it would be strengthened by addressing the comments below.

Major comments:

1. Figure 1 can be improved for visual clarity, particularly the following:

a. Timeline axis and date labels: Date formatting is inconsistent and some of the dates are difficult to read due to the dashed event stems running behind them. Please consider reformatting the timeline axis design to better illustrate the month and dates (please see this example: https://www.path.org/our-impact/articles/breaking-story-ebola-in-dr-congo/).

b. Event stems and markers: For some of the event markers (e.g., “DRC official declaration (175h Ebola outbreak)” it is unclear, to which event stem it is attached. Using branching lines on the event markers may help (again, please this example: https://www.path.org/our-impact/articles/breaking-story-ebola-in-dr-congo/).

c. Color coding: While the use of color codes for the detection gaps is helpful, the color coding of the event stems and markers do not appear to be consistent. For instance, the “Official alert notification” event stem corresponds to Event C and should be dark brown, but instead, I believe is colored the same as Event B. Additionally, for the events occurring after the last detection gap (i.e., after May 15), the use of color coding is not clear or intuitive. Please clarify the color coding of these events with a legend or keep them a uniform color (e.g., gray, similar to ‘27 May data lock’).

Minor comments:

• Line 114: Please spell out WASH (water, sanitation, and hygiene) at first use.

• Lines 130 (141, etc.): The phrase "public health emergency operation centre (INSP/COUSP)" is ambiguous; it’s unclear whether INSP and COUSP refer to separate institutions or a single entity. Please provide the full official names (in English and/or French, as appropriate) when first introduced and ensure the acronyms are used consistently throughout the manuscript.

• Lines 188-189: To improve clarity for PLOS GH readers not familiar with operational epidemiology, it may be helpful to provide a very brief definition of ‘detection gap’–something like “the elapsed time between successive milestones in outbreak recognition and response”–before jumping into the various intervals that were calculated.

• Lines 226-227: The manuscript states that alternative detection-gap definitions were evaluated using symptom onset, community signal, and official notification dates, but it is not clear which intervals were recalculated or which endpoint(s) remained fixed. A brief clarification would improve reproducibility.

• Line 288: Please remove the extra closed bracket “]”.

• Figure 2: Please consider replacing the forward slash in the y-axis title “Confirmed cases/deaths” to avoid potential confusion with division. Also please change “Nord- and Sud-Kivu” in the figure legend to North and South Kivu for consistent naming across the manuscript and figures.

• Line 486: There appears to be a track-change that needs to be resolved immediately before the in-text citation.

• S1 Table: Please clarify how the scoring thresholds (e.g., ≥10 confirmed cases, ≥20 suspected deaths, ≥200 contacts) were selected. Were these based on expert consensus, operational guidance, the observed distribution of the data, or another rationale? A brief description of the basis for these cutoffs would improve the transparency and reproducibility of the prioritization framework.

**********

what does this mean?). If published, this will include your full peer review and any attached files.

**Do you want your identity to be public for this peer review?**  If you choose “no”, your identity will remain anonymous but your review may still be made public.

For information about this choice, including consent withdrawal, please see our Privacy Policy

Reviewer #1: No

Reviewer #2: No

**********

[NOTE: If reviewer comments were submitted as an attachment file, they will be attached to this email and accessible via the submission site. Please log into your account, locate the manuscript record, and check for the action link "View Attachments". If this link does not appear, there are no attachment files.]

**Figure Resubmissions:**

Attachment Submitted filename: PGPH-D-26-02057-Reviewer1-CommentstoAuthors.docx

# Author comment: Author response to Decision Letter 1

**DOI:** 10.1371/journal.pgph.0006680.r002

23 Jul 2026

Attachment Submitted filename: Tambe Elvis Akem_Response_to_Reviewers.pdf

# Editor report: Decision Letter 1

**DOI:** 10.1371/journal.pgph.0006680.r003

**Authors:** Maria del Pilar Fernandez

3 Aug 2026

PGPH-D-26-02057R1

Operational epidemiology of the early phase of the 2026 Bundibugyo virus disease outbreak in the Democratic Republic of the Congo and Uganda

PLOS Global Public Health

Dear Dr. Elvis Akem,

Thank you for submitting your manuscript to PLOS Global Public Health. After careful consideration, we feel that it has merit but does not fully meet PLOS Global Public Health’s publication criteria as it currently stands. Therefore, we invite you to submit a revised version of the manuscript that addresses the points raised during the review process.

Please submit your revised manuscript by Sep 02 2026 11:59PM. If you will need more time than this to complete your revisions, please reply to this message or contact the journal office at globalpubhealth@plos.org. When you're ready to submit your revision, log on to https://www.editorialmanager.com/pgph/ and select the 'Submissions Needing Revision' folder to locate your manuscript file.

Please include the following items when submitting your revised manuscript:

- A letter that responds to each point raised by the editor and reviewer(s). You should upload this letter as a separate file labeled 'Response to Reviewers'.
- A marked-up copy of your manuscript that highlights changes made to the original version. You should upload this as a separate file labeled 'Revised Manuscript with Track Changes'.
- An unmarked version of your revised paper without tracked changes. You should upload this as a separate file labeled 'Manuscript'.

Guidelines for resubmitting your figure files are available below the reviewer comments at the end of this letter.

As the corresponding author, your ORCID iD is verified in the submission system and will appear in the published article. PLOS supports the use of ORCID, and we encourage all coauthors to register for an ORCID iD and use it as well. Please encourage your coauthors to verify their ORCID iD within the submission system before final acceptance, as unverified ORCID iDs will not appear in the published article. *Only*  the individual author can complete the verification step; PLOS staff *cannot*  verify ORCID iDs on behalf of authors.

We look forward to receiving your revised manuscript.

Kind regards,

Maria del Pilar Fernandez

Academic Editor

PLOS Global Public Health

**Journal Requirements:**

If the reviewer comments include a recommendation to cite specific previously published works, please review and evaluate these publications to determine whether they are relevant and should be cited. There is no requirement to cite these works unless the editor has indicated otherwise.

Please review your reference list to ensure that it is complete and correct. If you have cited papers that have been retracted, please include the rationale for doing so in the manuscript text, or remove these references and replace them with relevant current references. Any changes to the reference list should be mentioned in the rebuttal letter that accompanies your revised manuscript. If you need to cite a retracted article, indicate the article’s retracted status in the References list and also include a citation and full reference for the retraction notice.

**Additional Editor Comments (if provided):**

The authors have addressed the reviewer's comments in a satisfactory way. The only pending comment would be to strengthen the framing of the manuscript as an early-phase outbreak analysis tied to the stated data-lock dates. The manuscript should make clear that the case counts, operational indicators, health-zone prioritization, and interpretation reflect the public data available through the defined early analysis period, rather than the current or final status of the outbreak.

For example, please consider adding or revising language in the Abstract, Discussion, or Limitations along the following lines:

“This analysis reflects publicly available data from the early outbreak period, with the primary DRC data lock on 27 May 2026 and Uganda/regional triangulation through 29 May 2026. Because the outbreak and response continued beyond these dates, the findings should be interpreted as an early operational snapshot rather than a complete or current assessment of cumulative outbreak burden, geographic spread, or response performance.”

Please also review the conclusion to ensure that recommendations are framed as implications from this early operational snapshot, not as conclusions about the full outbreak trajectory.

[Note: HTML markup is below. Please do not edit.]

**Reviewers' comments:**

[NOTE: If reviewer comments were submitted as an attachment file, they will be attached to this email and accessible via the submission site. Please log into your account, locate the manuscript record, and check for the action link "View Attachments". If this link does not appear, there are no attachment files.]

**Figure Resubmissions:**

# Author comment: Author response to Decision Letter 2

**DOI:** 10.1371/journal.pgph.0006680.r004

3 Aug 2026

Attachment Submitted filename: Tambe_Elvis_Akem_Response_to_Reviewers.pdf

# Editor report: Decision Letter 2

**DOI:** 10.1371/journal.pgph.0006680.r005

**Authors:** Maria del Pilar Fernandez

12 Aug 2026

Operational epidemiology of the early phase of the 2026 Bundibugyo virus disease outbreak in the Democratic Republic of the Congo and Uganda

PGPH-D-26-02057R2

Dear Dr Elvis Akem,

We are pleased to inform you that your manuscript 'Operational epidemiology of the early phase of the 2026 Bundibugyo virus disease outbreak in the Democratic Republic of the Congo and Uganda' has been provisionally accepted for publication in PLOS Global Public Health.

Before your manuscript can be formally accepted you will need to complete some formatting changes, which you will receive in a follow up email. A member of our team will be in touch with a set of requests.

Please note that your manuscript will not be scheduled for publication until you have made the required changes, so a swift response is appreciated.

IMPORTANT: The editorial review process is now complete. PLOS will only permit corrections to spelling, formatting or significant scientific errors from this point onwards. Requests for major changes, or any which affect the scientific understanding of your work, will cause delays to the publication date of your manuscript.

If your institution or institutions have a press office, please notify them about your upcoming paper to help maximize its impact. If they'll be preparing press materials, please inform our press team as soon as possible -- no later than 48 hours after receiving the formal acceptance. Your manuscript will remain under strict press embargo until 2 pm Eastern Time on the date of publication. For more information, please contact globalpubhealth@plos.org.

Thank you again for supporting Open Access publishing; we are looking forward to publishing your work in PLOS Global Public Health.

Best regards,

Maria del Pilar Fernandez

Academic Editor

PLOS Global Public Health

***********************************************************

Reviewer Comments (if any, and for reference):
